# HLA frequency distribution of the Portuguese bone marrow donor registry

**DOI:** 10.3389/fimmu.2023.1286001

**Published:** 2023-12-12

**Authors:** Eduardo Espada, Dário Ligeiro, Hélder Trindade, João F. Lacerda

**Affiliations:** ^1^ Instituto de Medicina Molecular João Lobo Antunes, Faculdade de Medicina, Universidade de Lisboa, Lisbon, Portugal; ^2^ Serviço de Hematologia e Transplantação de Medula, Hospital de Santa Maria, Centro Hospitalar Universitário de Lisboa Norte, EPE, Lisbon, Portugal; ^3^ Centro de Sangue e Transplantação de Lisboa, Instituto Português do Sangue e da Transplantação, IP, Lisbon, Portugal; ^4^ Immunosurgery Unit, Centro Clínico Champalimaud, Lisbon, Portugal

**Keywords:** CEDACE, HLA, haplotype frequencies, Portugal, Portuguese-speaking African countries

## Abstract

**Introduction:**

The Portuguese donor Registry of CEDACE was the fifth largest *per capita* bone marrow donor Registry of the WMDA as of 2019 and has yet to be thoroughly analyzed. We aimed to characterize its various aspects, including demographics and HLA allele and haplotype frequencies, to evaluate the genetic matching propensity score and ultimately further develop it.

**Methods:**

We described and compared characteristics of the donor population with census data and used an Expectation-Maximization algorithm and analyses of molecular variance to assess haplotype frequencies and establish phylogenetic distances between regions and districts within the country.

**Results:**

We identified 396545 donors, corresponding to 3.85% of the Portuguese population; the median donor age was 39 years, with 60.4% of female donors. Most donors were Portuguese nationals, although 40 other nationalities were present, with a significant proportion of donors from Brazil and Portuguese-speaking African Countries; almost all donors self-reported as Western, with the second largest group reporting African ancestry. There was an asymmetric contribution of donors from different districts and regions, with most coming from coastal districts and few from the southern districts and autonomous regions; foreign and self-declared non-Western donors were mainly located in the Metropolitan Area of Lisbon and the South. Although most donors were typed in three *loci* (HLA-A, HLA-B and HLA-DRB1), only 44% were also typed in HLA-C, 1.28% in HLA-DQB1 and only 0.77% in all five *loci* and in high-resolution. There were varying allele and haplotype frequencies across districts and regions, with the most common three *loci*, low-resolution haplotypes, being HLA-A*01~B*08~DRB1*03, A*29~B*44~DRB1*07 and HLA-A*02~B*44~DRB1*04; some haplotypes were more prevalent in the South, others in the North and a few in the autonomous regions; African and foreign donors presented relevant differences in haplotype frequency distributions, including rare haplotypes of potential interest. We also report on four *loci*, low-resolution frequency distributions. Using AMOVA, we compared genetic distances between districts and regions, which recapitulated the country's geography.

**Discussion:**

Our analysis showed potential paths to optimization of the Registry, including increasing the male donor pool and focusing on underrepresented districts and particular populations of interest, such as donors from Portuguese-speaking African countries.

## 1 Introduction

The Human Leukocyte Antigen (HLA) genes, located in the major histocompatibility complex region in the short arm of chromosome 6, are a group of highly polymorphic genes involved in antigen presentation and T cell self-recognition that are preserved across generations, making them an appealing tool for assessing genetic differences and similarities between populations. Adequate HLA matching is still fundamental for unrelated hematopoietic cell transplantation, aiding in reducing the risk of acute and chronic graft-versus-host disease and graft rejection (1–6).

The CEDACE, an abbreviation of the National Center of Donors of Bone Marrow, Stem or Cord Blood Cells (*Centro Nacional de Dadores de Células de Medula Óssea, Estaminais ou de Sangue do Cordão*), was created in 1995 and includes the Portuguese bone marrow donor registry. It has multiple functions as described by the decree-law that generated its existence (*Despacho 22/95*), including organizing progenitor cell donor requests, coordinating progenitor cell donation, conservation and transplant activities, coordinating and organizing the recruitment and counseling of donors, and coordinating the HLA (Human Leukocyte Antigen) typing data of donors and keeping the aforementioned registry organized, among others. There are three Histocompatibility laboratories in Portugal, namely the Center for Blood and Transplantation of Lisbon (*Centro de Sangue e da Transplantação de Lisboa*), the Center for Blood and Transplantation of Porto (*Centro de Sangue e da Transplantação do Porto*), and the Center for Blood and Transplantation of Coimbra (*Centro de Sangue e da Transplantação de Coimbra*). CEDACE’s donor registry activities include communication with foreign registries, donors and donor centers, and transplantation and harvesting units to ultimately provide donor products to patients in need, regardless of country of origin (7).

Given the significant *per capita* size of the CEDACE registry, its composition in terms of foreign and ethnically diverse donors and the importance of its activities, we set out to characterize the Registry to fulfill three interconnected objectives: firstly, we aimed to evaluate the donor composition of the CEDACE registry in terms of demographics and distribution across the country; secondly, we attempted to describe and estimate HLA allele and haplotype frequencies of the CEDACE registry and subpopulations; and thirdly we intended to determine, using the results from the previous two objectives, which donor populations within the country should be targeted to further develop and improve the Registry.

## 2 Materials and methods

The CEDACE database was queried on August 8, 2017, to obtain epidemiological and HLA data for subsequent analysis. The World Marrow Donor Association (WMDA) website (8) was consulted on May 2, 2019, to obtain worldwide bone marrow donor registry sizes for comparison; the World Bank website (9) was consulted on the same day to calculate *per capita* registry sizes.

### 2.1 Descriptive analysis

Self-declared characteristics of the donor population obtained included age, gender, ethnicity/ancestry (described as “Origin”), nationality, and residence. Data from the 2011 national census (10) were used to compare with the Portuguese population. The statistical division in seven regions, using the Nomenclature of Territorial Units for Statistics (NUTS) II (North, Center, Metropolitan Area of Lisbon, Alentejo, Algarve, Autonomous Region of Azores and Autonomous Region of Madeira), and the administrative divisions in twenty districts (Açores, Aveiro, Beja, Braga, Bragança, Castelo Branco, Coimbra, Évora, Faro, Guarda, Leiria, Lisboa, Madeira, Portalegre, Porto, Santarém, Setúbal, Viana do Castelo, Vila Real and Viseu) and 308 municipalities were used for comparing subpopulations within the CEDACE registry and assess variations in donor distribution *per capita*. Graphical representations used maps adapted from http://d-maps.com (11, 12).

### 2.2 HLA frequency analysis

Information on HLA typing was collected for the five major *loci* used in matching for HCT (HLA-A, HLA-B, HLA-C, HLA-DRB1 and HLA-DQB1). The methods used for HLA data collection were sequence-specific oligonucleotide and sequence-specific primer for low- and intermediate-resolutions, whereas sequence-based typing was used for high-resolution data. After obtaining the raw database, empty cells corresponding to homozygous alleles were filled with the typing information from the other allele on the same *loci*, and one case of split antigen HLA-DR17 was converted into HLA-DRB1*03.

Several datasets were created based on the original raw data using the following sequence:

- Single letter referring to HLA typing resolution: L (low-resolution);
- Number of *loci* in the dataset: 3 (HLA-A/-B/-DRB1), 4 (HLA-A/-B/-C/-DRB1) and 5 (HLA-A/-B/-C/-DRB1/-DQB1);
- Descriptive of subpopulations within the dataset, if applicable: R (NUTS II Regions), D (districts), F (foreign donors), and NW (non-western donors). When no subpopulation was analyzed, the descriptive G (global) could be used.

As such, six low-resolution datasets, where the intermediate- and high-resolution data were converted into 2-digit, low-resolution data (e.g. HLA-DRB1*03:01 into HLA-DRB1*03) (13) were analyzed:

- Three *loci* (HLA-A, HLA-B and HLA-DRB1) datasets, dividing the donor populations by NUTS II Region and district (henceforth L3R and L3D, respectively – 394621 donors; L3G is derived from L3R); two datasets were also created analyzing foreign donors and donors with a self-declared non-western ancestry (L3F – 4022 donors – and L3NW – 2659 donors, respectively);
- Four *loci* (HLA-A, HLA-B, HLA-C and HLA-DRB1) and five *loci* (HLA-A, HLA-B, HLA-C, HLA-DRB1 and HLA-DQB1) datasets, divided by NUTS II Region, but mostly aimed at global donor database analysis (L4R – 174128 donors – and L5R – 5084 donors, respectively).

After the primary files were prepared with the assistance of CONVERT 1.3.1 (14), allele frequencies were calculated, HLA haplotype frequencies were estimated using the Expectation-Maximization algorithm in Arlequin ver. 3.5.2 (15), and exact tests of Hardy-Weinberg equilibrium were performed in the same software. Comparisons with the literature were then made with the assistance of the Allele Frequency Net Database (16), queried up to December 31, 2022. In the case of allele frequency data, gold standard population allele frequencies and the presence or absence of each frequent allele in typed populations were used to establish commonalities. In the case of haplotype frequency data, populations containing specific haplotypes with significant frequencies were considered if they comprised more than 1000 individuals, except in the case of rarer haplotypes, where all available evidence was used.

Assuming equal inter-allelic distances for all allele pairs, analyses of molecular variance (AMOVA) were also performed on Arlequin as a means to obtain a matrix of genetic distances between pairs of districts and regions at the low-resolution, three *loci* level, expressed by fixation indices (F_ST_). This matrix was then processed by MEGA 7.0.26 (17) to create neighbor-joining trees (18).

## 3 Results

The database obtained on August 14, 2017, comprised 396545 voluntary donors, corresponding to 3.85% of the Portuguese population. At the time, considering only bone marrow donor registries (excluding cord blood banks), CEDACE was the twelfth largest Registry in the world, and the fifth largest *per capita*, supplanted only by Cyprus, Israel, Germany, and Poland (**Supplementary Table 1**). Typing was performed at three CEDACE typing laboratories, with 46.01% of donors having been typed at the Center for Blood and Transplantation of Lisbon, 34.27% at the Center for Blood and Transplantation of Porto and 19.72% at the Center for Blood and Transplantation of Coimbra.

### 3.1 Descriptive analysis

#### 3.1.1 Global description

The median donor age in the CEDACE registry was 39 years (IQR 33-45). There was a predominance of female donors in the Registry (60.4% female; 39.6% male). Most donors in the Registry were of Portuguese nationality (98.98%), although 40 nationalities different than Portuguese were registered in CEDACE (**Table 1**). Brazil was the most represented country in the Registry other than Portugal, contributing 41.95% of foreign donors. When the Portuguese-speaking African Countries of Angola, Cape Verde, Guinea-Bissau, Mozambique, and São Tomé and Príncipe were added together, they contributed 30.48% of foreign donors. Of the 76.2% of donors who self-declared their ethnicity/ancestry, the vast majority (99.12%) reported being of Western origin; the second largest group (0.66%) reported African ancestry (**Supplementary Table 2**).

**Table 1:** Top: Countries of origin of donors represented in the CEDACE registry by at least 10 individuals and relative contribution. Bottom: Continents of origin of foreign donors in the CEDACE registry (Russia included in Europe).

Country of origin	Number of donors	Relative frequency
Portugal	392509	98.982%
Brazil	1693	0.427%
Cape Verde	601	0.152%
Angola	374	0.094%
France	241	0.061%
Ukraine	192	0.048%
Spain	137	0.035%
Mozambique	135	0.034%
Romania	79	0.020%
Germany	73	0.018%
Moldova	64	0.016%
Guinea-Bissau	61	0.015%
São Tomé and Príncipe	59	0.015%
United Kingdom	39	0.010%
Italy	39	0.010%
Poland	37	0.009%
South Africa	34	0.009%
Russia	33	0.008%
Netherlands	25	0.006%
Bulgaria	23	0.006%
Canada	20	0.005%
India	14	0.004%
United States	11	0.003%
Belgium	11	0.003%
Other	17	0.010%
Continent of origin	Number of donors	Relative frequency
South America	1708	42.32%
Africa	1271	31.49%
Europe	1005	24.90%
North America	31	0.77%
Asia	21	0.52%

#### 3.1.2 Geographical contribution

**Figure 1:**
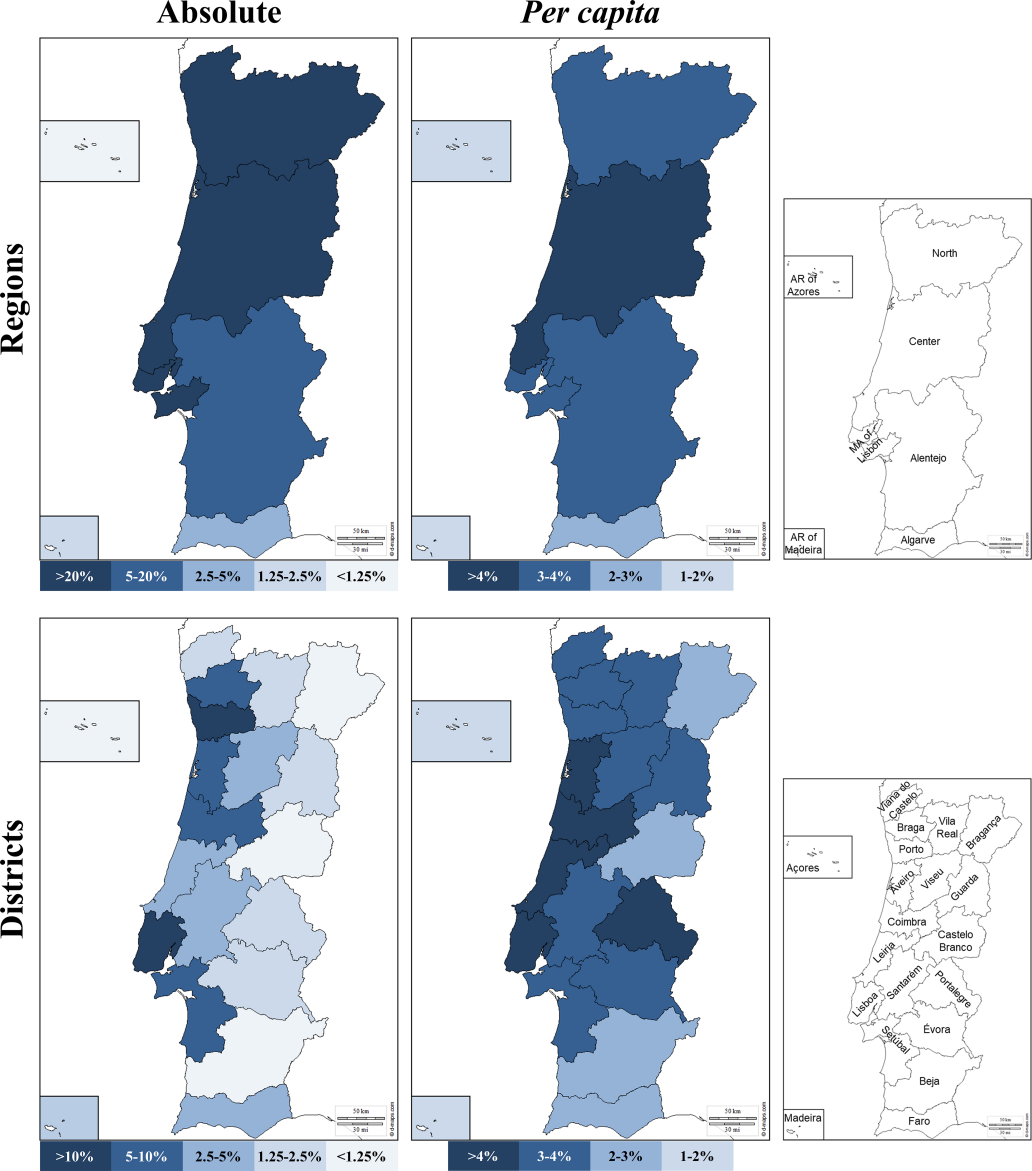
Donor NUTS II Region (top) and district of origin (bottom) in absolute (left) and *per capita* (right) contribution to the Registry. Maps adapted from http://d-maps.com.

**Figure 1**; **Supplementary Table 3** show the geographical distributions of donors in the CEDACE registry, both as absolute counts and *per capita* (relative to the region’s population), according to NUTS II Region and district of residence. Although most donors (87.4%) were provided by the NUTS II Regions of North, Metropolitan Area of Lisbon and Center, the differences between regions became less pronounced when *per capita* contributions were considered, with the Center Region contributing the most *per capita* donors and the Autonomous Regions of Madeira and Azores contributing the least. The five districts that contributed with the most donors were Lisboa, Porto, Braga, Aveiro and Setúbal; when considering *per capita* contributions, however, the five districts that contributed the highest percentage of donors were Coimbra, Portalegre, Aveiro, Leiria and Lisboa. The least represented districts regarding absolute contribution were Madeira, Castelo Branco, Açores, Bragança and Beja; and the five districts that contributed the least percentage of donors were Beja, Castelo Branco, Faro, Madeira and Açores. All municipalities but one had registered donors in the Registry, the exception being Corvo, with no registered donors; the highest *per capita* contributor municipalities were Vieira do Minho, Sobral de Monte Agraço and Murtosa, with 8.54%, 8.14% and 7.79%, respectively, and the lowest (except Corvo) were Santa Cruz das Flores, Lajes das Flores and Santa Cruz da Graciosa, with 0.35%, 0.33% and 0.26%, respectively – all belonging to the Autonomous Region of Azores. The five municipalities with the most donors were Lisboa, Sintra, Vila Nova de Gaia, Cascais and Oeiras, contributing to 5.26%, 3.84%, 3.27%, 2.40% and 2.01% of the Registry, respectively – of note, four of these belong to the Lisboa district and the Metropolitan Area of Lisbon Region.

The relative distribution of foreign donors and donors of self-declared non-western origin is represented in **Supplementary Figure 1**. The four regions that contributed with more foreign donors *per capita* were the Algarve, the Metropolitan Area of Lisbon, the Alentejo and the Autonomous Region of Azores, with 3.91%, 2.62%, 1.10% and 0.76%, respectively. In contrast, the Center, the Autonomous Region of Madeira and the North contributed with relatively few foreign donors, with 0.32%, 0.18% and 0.04%, respectively. Except for the Autonomous Regions, where the Azores had an unexpectedly higher percentage of foreign donors than Madeira, the frequency distribution relatively mimicked the distribution of foreign nationals residing in Portugal (10). In absolute terms, the Metropolitan Area of Lisbon contributed the vast majority of foreign donors (72.70%); it was followed by the Algarve, the Center, the Alentejo, the North, and the Autonomous Regions of Azores and Madeira, with 10.04%, 7.61%, 6.91%, 1.46%, 0.77% and 0.22%, respectively. The relative contribution of donors of self-declared non-western ethnicity shared some similarities with that of foreign donors, as it was higher in the South of the country (Metropolitan Area of Lisbon – 2.68%; Algarve – 1.66%; Alentejo – 0.65%), followed by the Autonomous Regions (Azores – 0.54%; Madeira – 0.18%) and lowest in the Center (0.15%) and North (0.02%). In absolute terms, most non-western donors resided in the Metropolitan Area of Lisbon (84.4%), followed by the Algarve, the Alentejo and the Center, with 5.09%, 4.68% and 4.49%. The North and the Autonomous Regions of Azores and Madeira contributed with a very low number of donors of non-western ethnicity, at 0.67%, 0.64% and 0.11%, respectively.

### 3.2 HLA frequency analysis

#### 3.2.1 Global description

As previously stated, the obtained database query consisted of 396545 donors; of these, 394621 (99.51%) were typed in at least three *loci* (HLA-A/-B/-DRB1), 174128 (43.91%) in at least four *loci* (including, besides the ones previously mentioned, HLA-C), and 5084 (1.28%) in all five *loci* (including HLA-DQB1). 3048 donors (0.77%) were typed in all five *loci* and intermediate to high-resolution (at the four-digit level), 63.71% of these with no ambiguities as expressed by NMDP codes, corresponding to 1942 donors, or 0.49% of the Registry. **Supplementary Tables 4**, **5** show the number of typed donors per NUTS II Regions and districts in each relevant dataset.

#### 3.2.2 Allele frequencies

Global low-resolution allele frequencies are shown in **Table 2**. As expected, HLA*B was the most polymorphic *locus*, followed by HLA*A; the least polymorphic *locus* was HLA-DQB1.

**Table 2:** Global low-resolution frequencies of HLA alleles. HLA-A, HLA-B and HLA-DRB1 frequencies obtained from L3R, HLA-C frequencies obtained from L4R and HLA-DQB1 frequencies obtained from L5R. Freq – frequency.

A	Freq	B	Freq	C	Freq	DRB1	Freq	DQB1	Freq
02	27.640%	44	14.806%	07	23.271%	07	16.160%	03	32.258%
01	10.920%	35	12.075%	04	15.654%	13	15.530%	02	22.335%
03	10.283%	51	10.317%	06	8.307%	04	13.006%	06	21.155%
24	10.178%	14	7.224%	05	7.674%	01	12.036%	05	20.024%
11	6.524%	08	6.718%	12	6.959%	03	10.972%	04	4.229%
29	5.434%	07	6.232%	16	6.805%	11	10.788%		
68	4.910%	15	5.548%	02	6.698%	15	8.266%		
23	4.492%	18	5.218%	03	6.539%	08	4.077%		
32	3.963%	49	3.516%	08	6.450%	14	2.732%		
33	3.431%	50	3.443%	15	5.194%	16	2.464%		
26	3.391%	40	3.407%	14	2.935%	10	1.716%		
30	2.972%	27	2.826%	01	2.269%	12	1.491%		
31	2.418%	57	2.692%	17	1.139%	09	0.761%		
25	1.361%	38	2.531%	18	0.106%				
66	0.864%	58	2.085%						
34	0.432%	39	1.606%						
69	0.339%	13	1.488%						
74	0.218%	45	1.335%						
36	0.117%	53	1.282%						
80	0.113%	37	1.279%						
43	0.001%	41	1.149%						
		55	1.149%						
		52	0.880%						
		56	0.371%						
		47	0.312%						
		42	0.156%						
		78	0.156%						
		48	0.072%						
		73	0.057%						
		81	0.026%						
		67	0.023%						
		46	0.010%						
		82	0.006%						
		54	0.004%						
		59	0.001%						

The most frequent HLA*A alleles found in the CEDACE registry were the common alleles HLA-A*02 and HLA-A*24, as well as the typically Western alleles HLA-A*01 and HLA-A*03, with a cumulative frequency of 59% of the identified low-resolution HLA-A polymorphisms. While it was shown to be a more polymorphic *locus*, the five most frequent HLA-B alleles (the common HLA-B*44, HLA-B*35, HLA-B*51 and HLA-B*08, and the Western/African HLA-B*14) cumulatively represented more than 50% of the identified low-resolution polymorphisms. Some rare alleles, such as the East Asian HLA-B*46, HLA-B*54 and HLA-B*59 or the Sub-Saharan African HLA-B*82, could be detected at very low frequencies in our Registry. The two common HLA*C alleles, HLA-C*07 and HLA-C*04 had a cumulative frequency of 39%; the rarest identified HLA-C allele is the Sub-Saharan African HLA-C*18, with a frequency of 0.106%. There were six HLA-DRB1 alleles with a detected frequency higher than 10%, comprising 78.5% of detected HLA-DRB1 alleles: the Western/African HLA-DRB1*07, HLA-DRB1*13 and HLA-DRB1*01, and the common HLA-DRB1*04, HLA-DRB1*03 and HLA-DRB1*11. The three HLA-DRB1 alleles identified at the lowest frequencies were the typically Asian alleles HLA-DRB1*10, HLA-DRB1*12 and HLA-DRB1*09. The five low-resolution HLA-DQB1 alleles were identified in the CEDACE registry, four of them with a cumulative frequency higher than 20%; the only HLA-DQB1 allele that was found with a lower frequency was HLA-DQB1*04 (16).

##### 3.2.2.1 Distribution of allele frequencies by NUTS II Region

Allele and HLA frequencies varied according to districts and NUTS II Regions of residence, as well as ancestry and nationality of donors. Differential low-resolution allele distributions according to NUTS II Region are demonstrated in **Supplementary Figures 2-6**, after a brief description in the Supplementary data file (datasets L3R, L4R and L5R).

#### 3.2.3 Haplotype frequencies

HLA haplotype frequency distribution in the CEDACE registry was skewed towards more frequent haplotypes. In fact, in the CEDACE registry at the three *loci*, low-resolution level, there was a significant deviation from Hardy-Weinberg equilibrium on all three *loci* (p-value<0.00001). Due to these factors, the most frequent haplotypes identified comprised a relatively high fraction of them.

##### 3.2.3.1 In the CEDACE registry, NUTS II Regions and districts

###### 3.2.3.1.1 Three *loci*, low-resolution haplotype frequencies

A total of 4913 three *loci*, low-resolution HLA haplotypes with a frequency of at least 0.0001% were identified in the CEDACE registry (L3G). Of these, the five most common corresponded to 8.85% of the Registry, the ten most common to 13.82%, the 25 most common to 23.50%, the 50 most common to 31.96%, the 100 most common to 42.25%, the 150 most common to 50.03%, the 500 most common to 76.31%, and the 500 most common to 89.47%. For the 394621 donors identified in L3G, there were 167505 individual genotypes, leading to 227116 individuals, or 57.55%, having at least one other matched individual in the CEDACE registry at this resolution.

**Figure 2:**
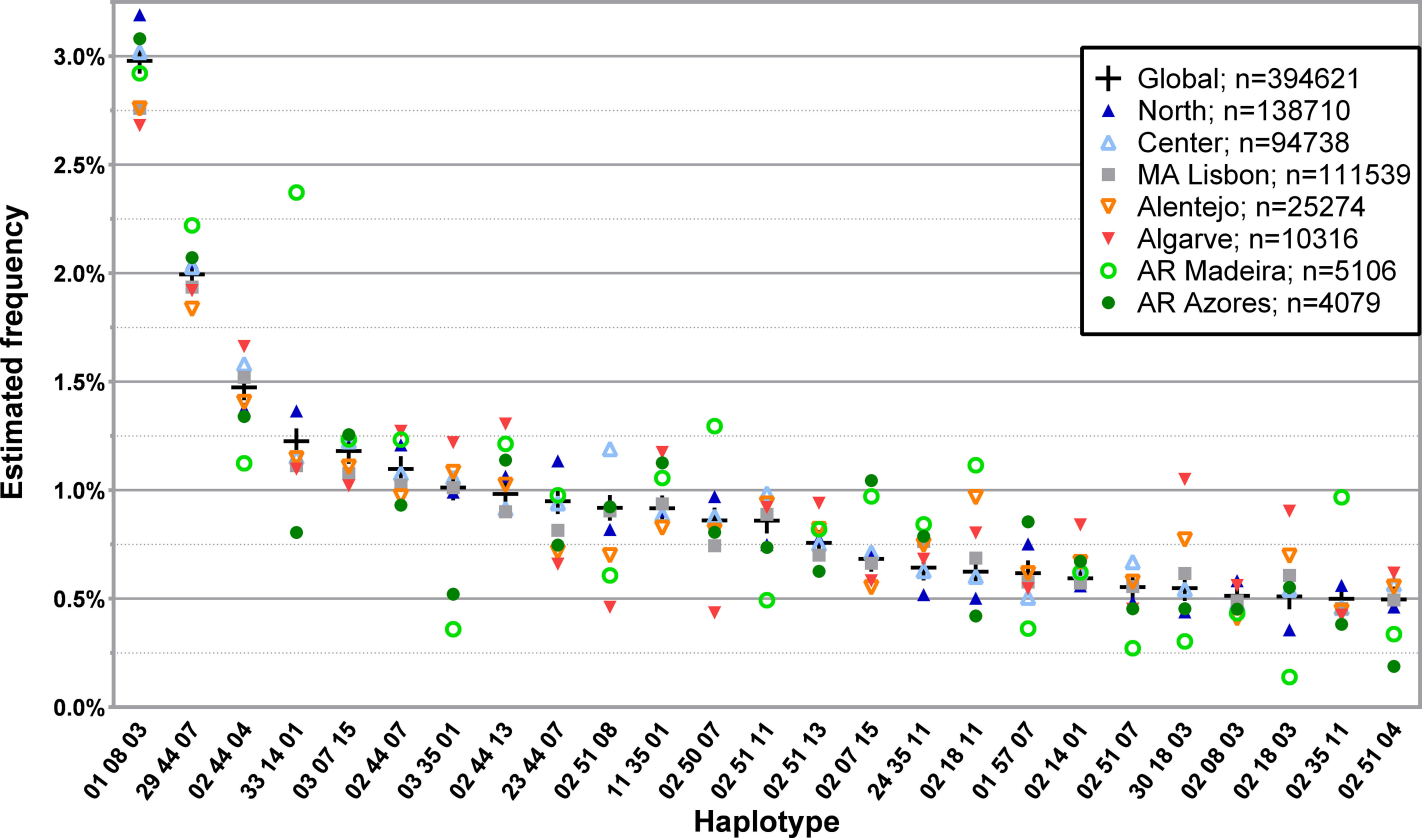
Frequency distribution of the 25 most common three *loci*, low-resolution HLA-A/-B/-DRB1 haplotypes in CEDACE and corresponding frequencies according to NUTS II Region. Dataset: L3R.

The frequency distribution of the 150 most common three *loci*, low-resolution haplotypes identified in the CEDACE registry (L3G) and corresponding frequencies according to NUTS II Region (L3R) are displayed in **Supplementary Table 6**, and a graphical representation of the frequency distribution of the 25 most common haplotypes is shown in **Figure 2**.

Considering the most frequent L3G haplotypes, some displayed increasing frequencies from south to north, such as HLA-A*01~B*08~DRB1*03, and some from north to south, such as HLA-A*02~B*18~DRB1*11, HLA-A*30~B*18~DRB1*03 and HLA-A*02~B*18~DRB1*03. Specific haplotypes had greater frequencies in the Autonomous Regions, such as HLA-A*29~B*44~DRB1*07, detected with a greater relative frequency in the Autonomous Region of Madeira, followed by the Autonomous Region of Azores. Similarly, HLA-A*33~B*14~DRB1*01 and HLA-A*02~B*35~DRB1*11 were seen in the Autonomous Region of Madeira with a much higher frequency than in the CEDACE registry (2.3724% vs. 1.2262% and 0.9684% vs. 0.4992%, respectively). HLA-A*03~B*35~DRB1*01, on the contrary, while frequent in the CEDACE registry (1.0120%), was much less frequently found in the Autonomous Regions of Madeira (0.3593%) and Azores (0.5214%).

The frequency distribution of the 150 most common haplotypes in L3G according to District (L3D) is displayed in **Supplementary Tables 7**, **8**, and a graphical representation of the frequency distribution of the 25 most common haplotypes is shown in **Supplementary Figure 7**.

###### 3.2.3.1.2 Four *loci*, low-resolution haplotype frequencies

**Figure 3:**
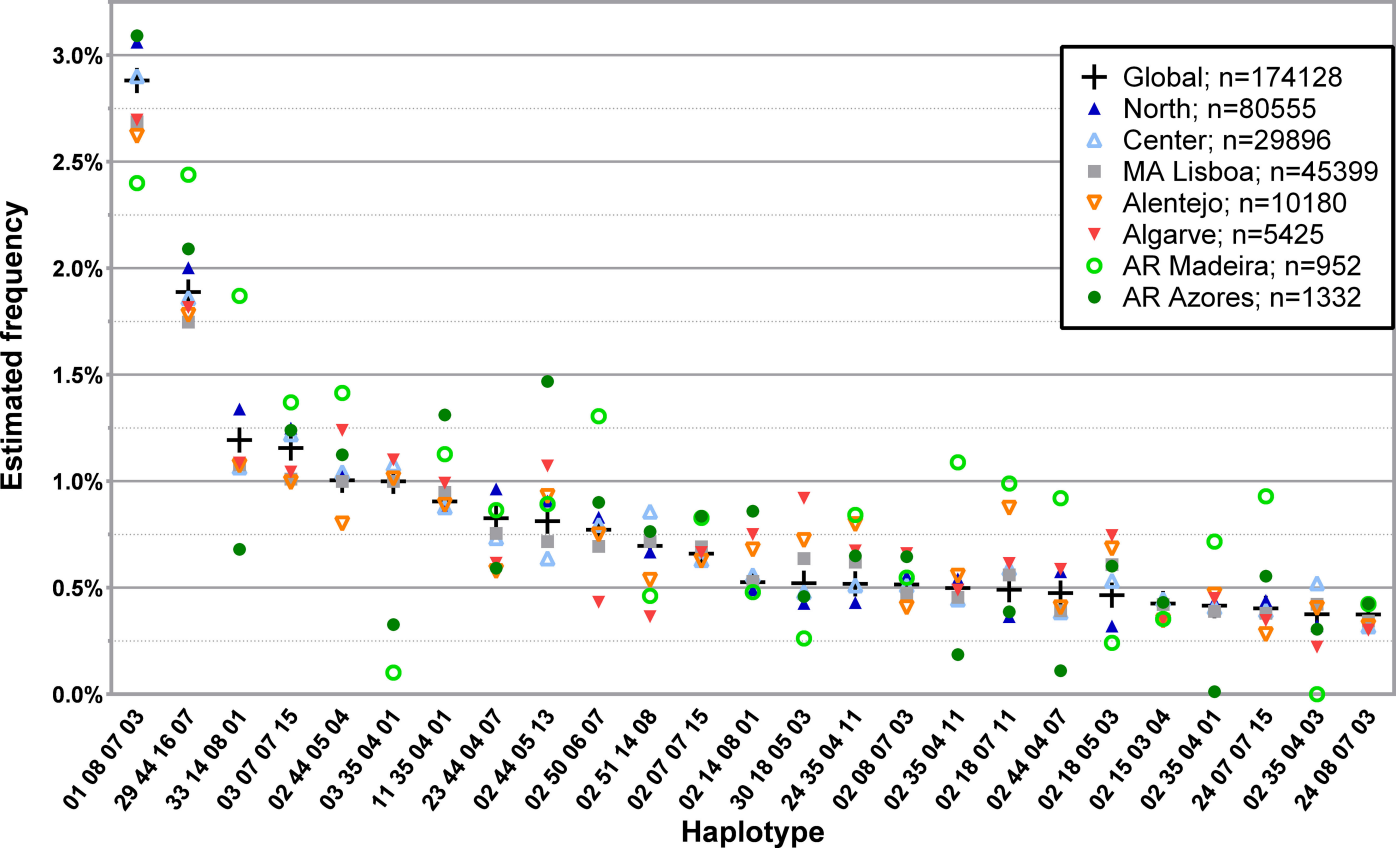
Frequency distribution of the 25 most common four *loci*, low-resolution HLA-A/-B/-C/-DRB1 haplotypes in CEDACE and corresponding frequencies according to NUTS II Region. Dataset: L4R.

The analysis of L4R led to the identification of 9627 individual four *loci*, low-resolution haplotypes with a frequency of at least 0.0001%. For the 174128 donors in this database, there were 119196 individual genotypes, leading to 54932 individuals, or 31.55%, having at least one other matched individual in the CEDACE registry. The frequency distribution of the 25 and 50 most common four *loci*, low-resolution haplotypes in the CEDACE registry and according to NUTS II Region is represented in **Figure 3**; **Supplementary Table 9**, respectively.

###### 3.2.3.1.3 Neighbor-joining trees

**Figure 4:**
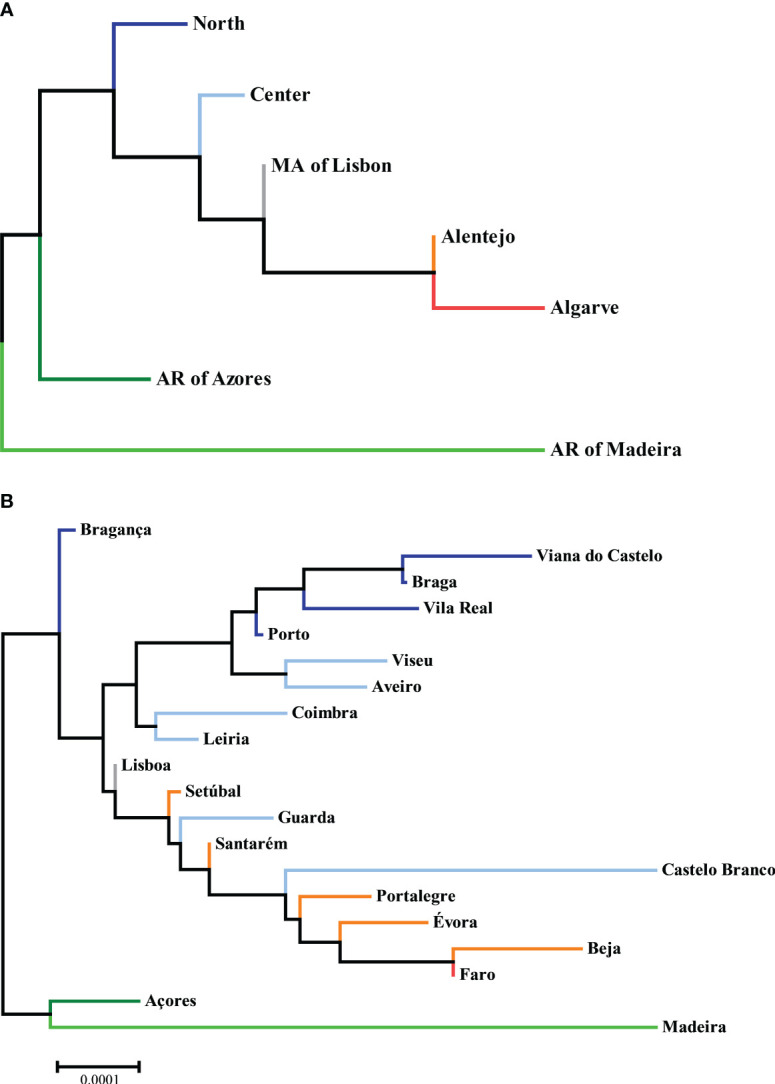
Neighbor-joining trees of phylogenetic distances between **(A)** NUTS II Regions and **(B)** Districts, with the sum of branch lengths of 0.00150375 and 0.00346773, respectively, drawn to scale. Datasets: L3R and L3D.

Using the AMOVA function, as previously described, we obtained matrices of F_ST_, reflecting the genetic distances between the donor populations of different NUTS II Regions and Districts, as shown in **Supplementary Figures 8**, **9** and displayed in graphical format in **Figure 4**. All pairwise comparisons were highly statistically significant, with p-values <0.00001.

Regarding NUTS II Regions, one can appreciate that the ones more similar to the global CEDACE registry (and thus more reflective of overall contribution to itself) were the Metropolitan Area of Lisbon and the Center, whereas the one more unlike it was the Autonomous Region of Madeira, followed by the Algarve and the Autonomous Region of Azores. Regarding Districts, the closest one to the CEDACE registry was Lisboa, followed by Porto and Leiria, Santarém and Setúbal, and the one furthest from it was Madeira, followed by Castelo Branco, Beja, Évora and Faro.

##### 3.2.3.2 In African and foreign donors

**Figure 5:**
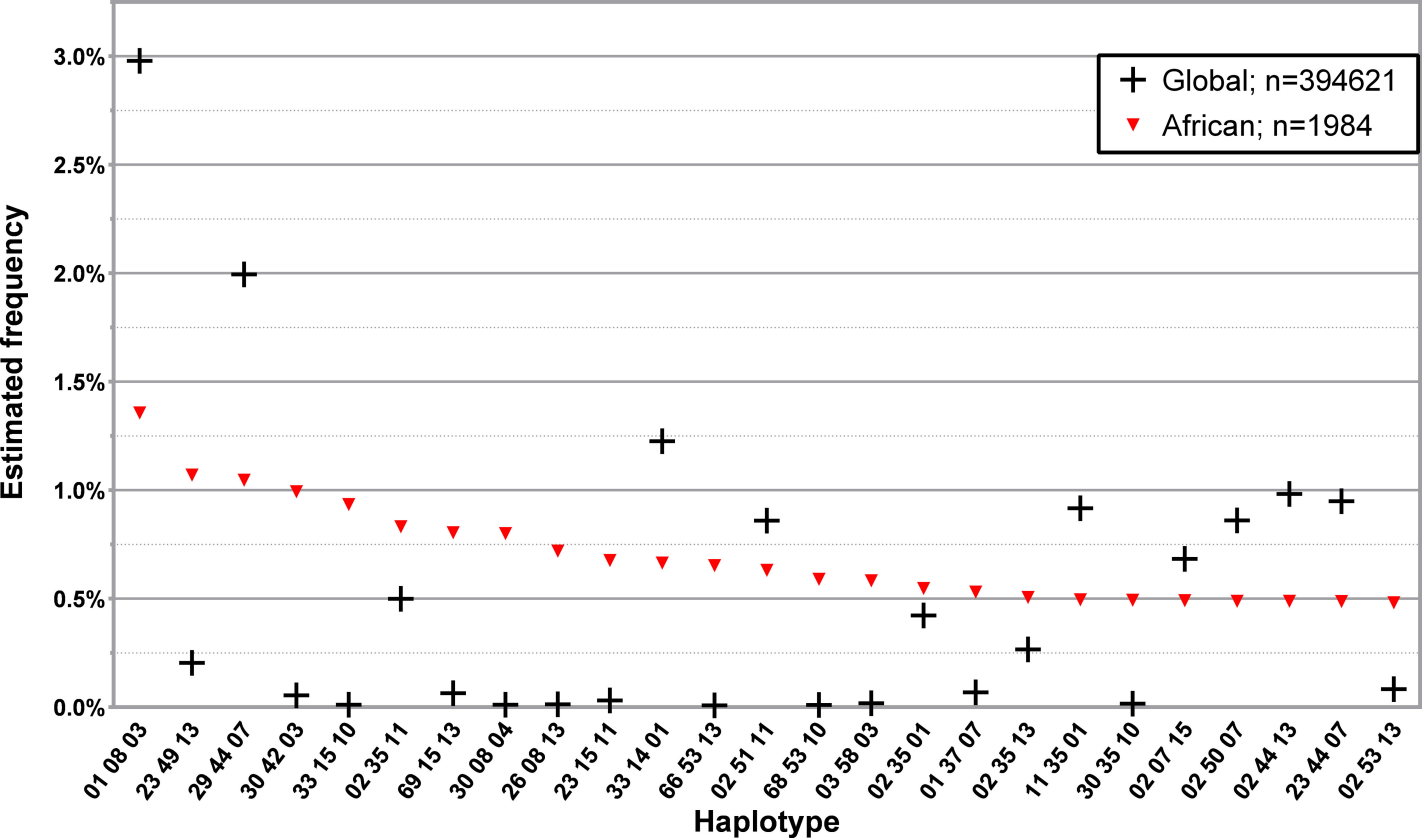
Frequency distribution of the 25 most common three *loci*, low-resolution HLA-A/-B/-DRB1 in donors of self-declared African ancestry. Dataset: L3NW.

Due to the low number of non-western donors in the Registry, only the African subset of L3NW was specifically analyzed. The 25 and 50 most common haplotypes found in the 1984 donors with self-declared African ancestry and the comparison with the corresponding L3G haplotype frequencies are represented in **Figure 5**; **Supplementary Table 10**, respectively. While the first and third most frequent three *loci*, low-resolution haplotypes in this donor group (HLA-A*01~B*08~DRB1*03 and HLA-A*29~B*44~DRB1*07, respectively), were the first and second most frequently identified haplotypes in the CEDACE registry, there were several haplotypes detected with significant frequency (greater than 0.5%) in this population that had comparatively low frequencies in the CEDACE registry.

Examples of this are HLA-A*23~B*49~DRB1*13, HLA-A*30~B*42~DRB1*03 and HLA-A*33~B*15~DRB1*10, the second, fourth and fifth most frequent haplotypes in African donors, which had a frequency of 1.0707%, 0.9937% and 0.9348% in this donor population and 0.2041%, 0.0547% and 0.0112% in the CEDACE registry, respectively.

**Figure 6:**
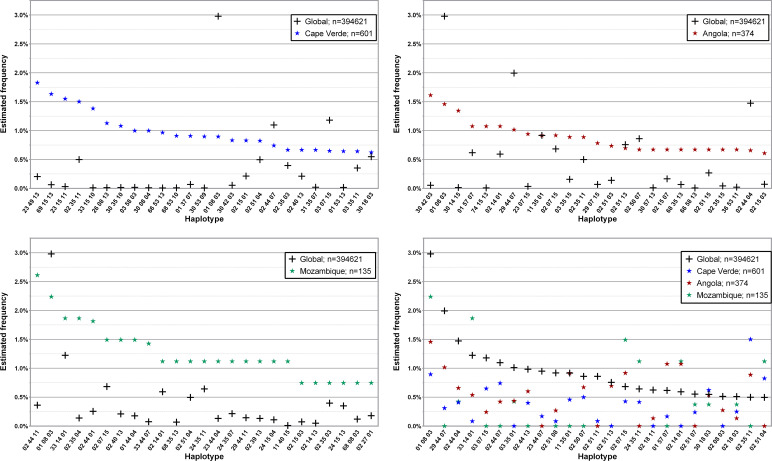
Frequency distribution of the 25 most common three *loci*, low-resolution HLA-A/-B/-DRB1 in donors from the three Portuguese-speaking African countries compared to the global CEDACE Registry. Top left: Cape Verde; top right: Angola; bottom left: Mozambique; bottom right: CEDACE and all three countries. Dataset: L3F.

Similarly to what was done in regards to L3NW, only a few donor populations from L3F were analyzed, according to historical interest, available evidence and donor population size. **Figure 6**; **Supplementary Tables 11**–**13** show the 25 and 50 most common three *loci*, low-resolution haplotypes found in the 601, 135 and 374 donors with self-declared nationalities from the Portuguese-speaking African Countries (PALOP) with more than 100 registered donors: Cape Verde, Mozambique and Angola, respectively. Donors from Cape Verde (disregarding 7.3% missing data) were mainly (87.8%) of self-declared African ancestry, with 8.4% self-describing as Western and 3.6% as Mixed ancestry. Of the 134 donors from Mozambique in the CEDACE registry, only 46.3% self-declared their origin; of these, 48.4% reported Western ancestry, 40.3% African ancestry, and 9.7% Mixed ancestry (one donor reported Hindu ancestry). Only 206 out of the 372 (55.4%) donors from Angola declared their origin: 69.9% self-declared African ancestry, 23.3% Western, and 6.8% Mixed.

**Figure 7:**
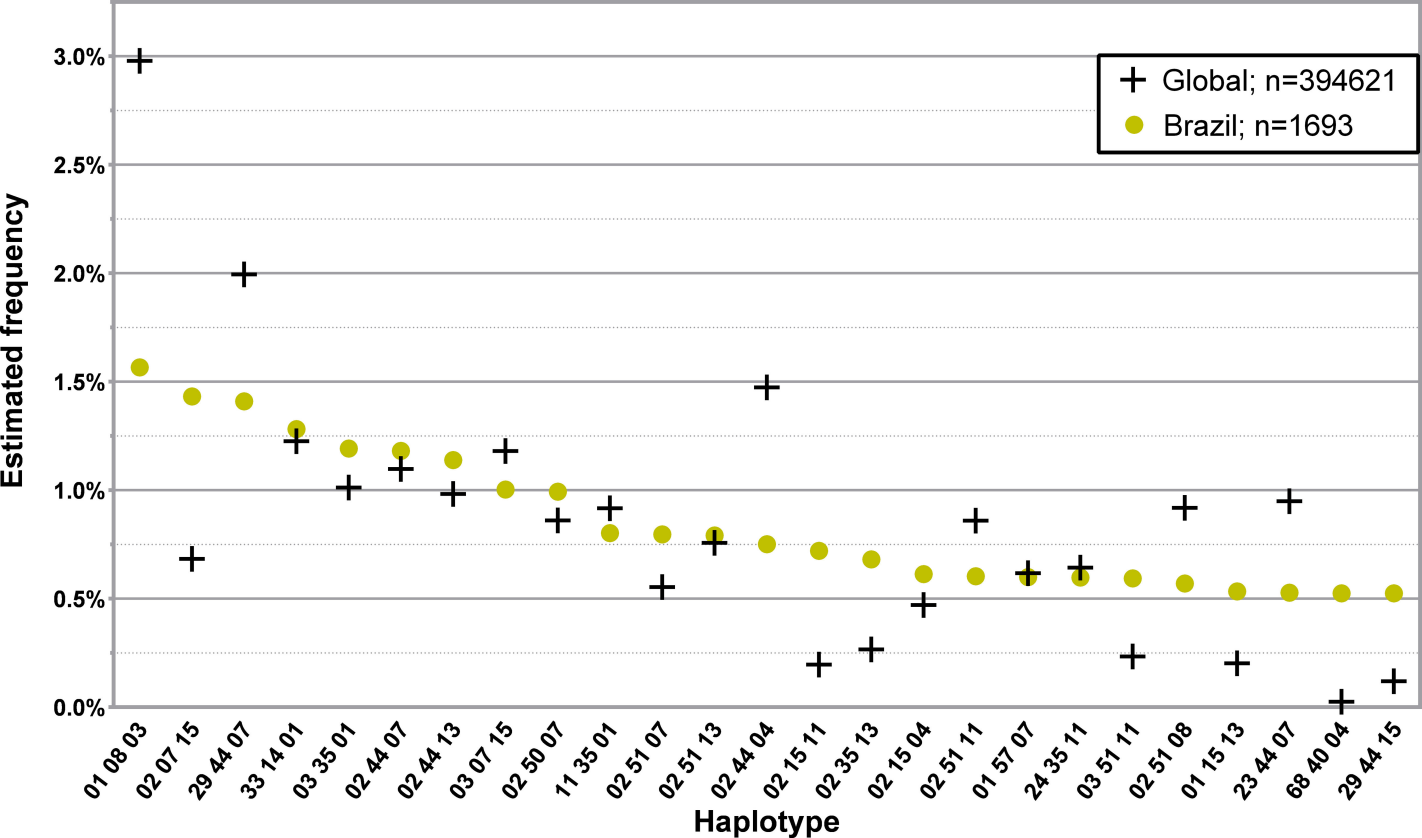
Frequency distribution of the 25 most common three *loci*, low-resolution HLA-A/-B/-DRB1 in donors from Brazil. Dataset: L3F.

The 25 and 50 most common three *loci*, low-resolution haplotypes in the Brazilian donor population of the CEDACE registry are displayed in **Figure 7**; **Supplementary Table 14**, respectively. Of the 81.1% who self-declared their ancestry, 89.1% were Western, 8.0% Mixed, 1.5% African and 0.8% Asian.

##### 3.2.3.3 Hardy-Weinberg equilibrium

**Supplementary Table 15** highlights significant deviations from Hardy-Weinberg equilibrium in L3R, L3D, L4R, and in the analyzed subpopulations of L3NW and L3F.

In L3R, there was no significant deviation from Hardy-Weinberg equilibrium on any of the three *loci* in Alentejo and Algarve; there was significant deviation from Hardy-Weinberg equilibrium on all three *loci* in the Metropolitan Area of Lisbon and the Center, only on HLA-A and HLA-B in the North, only on HLA-A in the Autonomous Region of Madeira, and on HLA-B and HLA-DRB1 in the Autonomous Region of Azores. Regarding L3D, there was no significant deviation from Hardy-Weinberg equilibrium on any of the three *loci* in Viana do Castelo, Vila Real, Bragança, Castelo Branco, Évora, Beja and Faro; there was significant deviation from Hardy-Weinberg equilibrium on all three *loci* in Leiria, Lisboa and Setúbal, only on HLA-A in Madeira, only on HLA-B in Viseu, Guarda, Coimbra and Portalegre, on HLA-A and HLA-B in Porto, on HLA-A and HLA-DRB1 in Santarém, and on HLA-B and HLA-DRB1 in Braga, Aveiro and Açores.

In L4R, there was no significant deviation from Hardy-Weinberg equilibrium on any of the four *loci* in Alentejo, Algarve and the Autonomous Region of Madeira; there was significant deviation from Hardy-Weinberg equilibrium on all four *loci* in the Global analysis of the CEDACE registry and the Metropolitan Area of Lisbon, only on HLA-A and HLA-DRB1 in the Center and the North, and only on HLA-DRB1 in the Autonomous Region of Azores.

Regarding the population of African donors in L3NW, there was significant deviation from Hardy-Weinberg equilibrium on HLA-A and HLA-B. Finally, for the analyzed subpopulations of L3F, no significant deviations from Hardy-Weinberg equilibrium were found for any *locus*.

## 4 Discussion

### 4.1 Descriptive analysis

This is the first extensive analysis of HLA allele and haplotype frequencies of the CEDACE registry, one of the world’s largest *per capita* bone marrow donor registries. The general characterization herein presented is functional because it provides data on the composition of the Registry and allows for correlation with the Portuguese resident population. Namely, we demonstrated a predominance of female donors in the Registry, which was more expressive than the gender difference in the country: where the CEDACE registry contained 60.4% female donors, there were 52.6% female residents in the 2011 Census (10). We also showed a smaller relative representation of foreign nationals in the donor registry (1.02%) than the population residing in the country (3.49%) (10), noting the significant contribution of donors from Brazil and the Portuguese-speaking African countries. Although Portuguese census data lack ethnicity and ancestry data, the fact that more than 99% of donors who self-reported their ancestry identified as “Western” suggests a skew in the ethnic composition of the Registry, which, coupled with the knowledge of the gender imbalance and low representation of foreign nationals, provide a first step towards potential optimization of CEDACE, via targeted donor recruitment campaigns. Regarding the absolute and relative distributions of donors according to district and NUTS II Region, the distribution of donors suggests that ease of access to typing laboratories (North in Porto, Center in Coimbra and South in Lisbon) seems to be directly related to the relative contribution of donors to the Registry, with more remote locations consistently contributing fewer donors. Other reasons for this imbalance may be related to increased access to donor drives and better information resource availability in big cities, particularly those with large college communities, allowing for greater recruitment in these areas.

### 4.2 HLA frequency analysis

The most frequent three *loci*, low-resolution haplotype in the CEDACE registry was HLA-A*01~B*08~DRB1*03, which has been found at a frequency above 5% in populations of the British Isles, including populations of individuals of United Kingdom descent in the German Registry (19–21), American Caucasoid populations (22, 23) and Northern European populations, including those in the Polish and German Registries (21, 24, 25). The second most frequent haplotype identified in our Registry was HLA-A*29~B*44~DRB1*07; it has been found with frequencies greater than 2.5% in populations of Spain (21, 26) and the Caribbean (22). The third most commonly identified haplotype was HLA-A*02~B*44~DRB1*04, previously found in frequencies greater than 1.5% in populations of the British Isles (19–21) and Caucasoid populations in the American Registry (22, 23). HLA-A*33~B*14~DRB1*01, more predominant in the Autonomous Region of Madeira, has been previously found with frequencies greater than 2% in Jewish populations in the Israeli Registry, namely with self-declared Kavkazi, Argentinian, Ashkenazi, Polish, American and Russian descent (27), adding to the evidence of Jewish ancestry being prevalent in this region (28). The fifth most common haplotype was HLA-A*03~B*07~DRB1*15, commonly identified in Caucasoid populations of the NMDP registry (22) and northern European minority populations of the DKMS registry (21).

Disregarding a previous unpublished analysis of the CEDACE registry, HLA-A*02~B*44~DRB1*07, which had a frequency of 1.0981% in our Registry, has only been found with a relative frequency above 1% in populations larger than 1000 individuals in the Portuguese minority of the DKMS (21), in blood donors in Wales (19) and in the Jewish population from Lybia in the Israeli Registry (27). HLA-A*03~B*35~DRB1*01, commonly found in Russia, Norway, Germany and Austria (16, 21, 24, 29), was more frequent in continental Portugal than in the Autonomous Regions. HLA-A*02~B*50~DRB1*07, less common in the Algarve (0.4353%) and more common in the Autonomous Region of Madeira (1.2959%) than in the CEDACE registry (0.8603%) is another haplotype more commonly found in Jewish populations of the Israeli Registry, namely from Lybia, Tunisia, United States of America, Argentina and Poland, and the Druze minority (27).

HLA-A*02~B*18~DRB1*11, HLA-A*30~B*18~DRB1*03 and HLA-A*02~B*18~DRB1*03 were all found to have increasing relative frequencies from north to south. The first has previously been described with relative frequency over 1.5% in the Macedonian and Croatian Registries (30, 31), as well as in minorities from Greece, Croatia, Bosnia and Herzegovina and Romania in the DKMS registry (21) and in Kavkazi and Druze populations of the Israeli Registry (27); the second one with relative frequency over 1.5% in large populations in Spain and the Spanish minority of the DKMS (21, 26, 32–34); and the third one has only been described with a frequency above 0.5% in our previous unpublished analysis, in an unpublished analysis of the Spanish Registry (16), as well as small population reports in Mexico and Brazil (35, 36). Of note, HLA-A*02~B*35~DRB1*11, the 24^th^ most common haplotype in the CEDACE registry, more commonly found in the Autonomous Region of Madeira, has only been described with a relative frequency over 1% in populations of Macedonia (30), Mexico (35), Iran (37), Albania (38), Gaza (39), Jordan (40), Israel (27), and a small population study in Guinea Bissau and Cape Verde (41).

Regarding the haplotype frequencies observed in African donors, it is important to note that certain haplotypes have only been described with relevant frequencies in small population studies. HLA-A*23~B*49~DRB1*13, for instance, has only been described with a frequency above 1% in two small studies, appearing in a small sample of Portuguese volunteers (46 individuals) from the North of Portugal (42) and in 62 individuals from the Northwest of Cape Verde (41); it has also been described with significant frequency (0.6190%) in Ethiopian Jews from the Israeli Registry (27). HLA-A*30~B*42~DRB1*03, a haplotype found to be 18 times more frequent in the African donor population of the CEDACE registry, when compared to the whole Registry, has been described with significant frequency in a small study of 202 unrelated blood donors from Mozambique (43), as well as short population reports from Kenya (44), Brazil (45), South Africa (46) and the United Arab Emirates (47) and African American individuals in the NMDP registry (22). HLA-A*33~B*15~DRB1*10, which had a frequency 83.5 times higher in the African donor population, was previously identified with a relative frequency above 0.5% only in populations of Guinea Bissau and Cape Verde (41). The sixth and seventh most frequent haplotypes in this population, HLA-A*69~B*15~DRB1*13 and HLA-A*30~B*08~DRB1*04, with frequencies of 0.8043% and 0.8008% (12.4 and 67.9 times more frequent than in the CEDACE registry) have only been identified with frequencies above 0.2% in the same previously cited study, in populations of Cape Verde (41) and, in the case of HLA-A*30~B*08~DRB1*04, in Mexico (35).

Regarding HLA frequencies of donors from Cape Verde, of the 25 most commonly identified haplotypes, only 9 were previously described in the largest study of Cape Verde nationals, which contained roughly one fifth of the population in our Registry (41). Of note, HLA-A*66~B*53~DRB1*13 and HLA-A*68~B*53~DRB1*10 have never been described in populations with frequencies above 0.5% (16) and were identified in this population with frequencies of 0.9659% and 0.9103%, respectively (120.7 and 90.1 times the respective frequencies in the CEDACE registry). Of the 25 most commonly identified 3 *loci*, low-resolution HLA haplotypes in the population of donors from Mozambique, only one, HLA-A*29~B*44~DRB1*11, was also reported among the 15 most common haplotypes in the most extensive study of Mozambican natives (43). The most commonly identified haplotype in the population of Angolan donors, HLA-A*30~B*42~DRB1*03, was the most frequently identified haplotype in the previously mentioned study of blood donors from Mozambique (43), as well as the fourth most commonly identified haplotype among self-declared African donors. There is a scarcity of literature regarding HLA haplotype frequencies of Angolan natives; our analysis is, to our knowledge, the first provider of HLA haplotype frequency data in this population.

We presented the most common haplotypes in the Brazilian donor population of the CEDACE registry because it is the largest foreign donor population in our Registry. In general, haplotype frequencies varied when compared to CEDACE, but frequent haplotypes were generally similar. A noteworthy exception is HLA-A*68~B*40~DRB1*04, with a frequency 20.9 times higher in this population than in CEDACE, which has mostly been described in indigenous populations of Mexico (35), as well as Costa Rica, Nicaragua (48), Venezuela (49) and Guatemala (50).

### 4.3 Limitations and future directions

One of the limitations of our study was the widespread significant deviation from Hardy-Weinberg equilibrium observed in most populations. This may induce errors in the Estimation-Maximization algorithm, although is an expected phenomenon, commonly found in large datasets, such as donor registries, which can be attributed, among other causes, to non-random selection and migration (51). One aspect of our analysis that may serve to partially validate the results is that the neighbor-joining trees seem to reasonably recapitulate the geographical distances between Regions and Districts, even though the CEDACE registry is not a random sample of the Portuguese population. Its analysis can therefore not be fully equated to an analysis of Portugal as a whole.

Another critical limitation of our study is the low resolution of the HLA data presented, especially when current guidelines recommend high-resolution typing for patient-donor matching (52, 53). This stemmed from the fact that the vast majority of the donors in the CEDACE registry were typed on intermediate or low-resolution and no extrapolation from the limited number of donors typed in high-resolution was possible – only less than 2000 donors were typed in high-resolution and this typing was biased, since all the high resolution data, at the time of data collection, was obtained through retyping of donors done after activation to match patients. Since 2020-2021 typing for all new donors has been done using next-generation sequencing, including typing at the DPB1 *locus*, warranting a new analysis and comparison with the low-resolution haplotype frequencies herein presented after sufficient new donors have been typed using this method.

With the information presented in this study, we propose targeting specific groups within Portugal’s borders to optimize the Registry. Namely, targeting under-represented districts (*per capita*), such as Beja, Bragança, Castelo Branco and Açores, or genetically more distinct from the Registry, such as Madeira, Castelo Branco and, again, Beja, would increase the diversity of the Portuguese donor pool. Considering the significant proportion of foreign residents from Portuguese-speaking African Countries, as well as these countries’ historical, economic, and social ties to Portugal, the fact that there are no bone marrow donor registries in these countries and the diverse HLA haplotypes found in donors from these countries as well as self-declared African donors, it would be advantageous to the Registry and African patients to include more donors from these groups.

## Data availability statement

The original contributions presented in the study are included in the article/**Supplementary Material**. Further inquiries can be directed to the corresponding author.

## Ethics statement

Ethical approval was not required for the study involving humans in accordance with the local legislation and institutional requirements. Written informed consent to participate in this study was not required from the participants or the participants’ legal guardians/next of kin in accordance with the national legislation and the institutional requirements.

## Author contributions

EE: Conceptualization, Formal analysis, Funding acquisition, Investigation, Methodology, Project administration, Visualization, Writing – original draft, Writing – review & editing. DL: Conceptualization, Data curation, Project administration, Writing – review & editing. HT: Conceptualization, Project administration, Writing – review & editing. JL: Conceptualization, Project administration, Supervision, Validation, Visualization, Writing – original draft, Writing – review & editing.
