# Supplementary material for: HLA frequency distribution of the Portuguese bone marrow donor registry
Source: Front Immunol. 2023 Dec 12;14:1286001. doi: 10.3389/fimmu.2023.1286001 (PMC10749969; doi:10.3389/fimmu.2023.1286001)
Supplement: Supplementary file 1 [file DataSheet_1.pdf]

Supplementary material

Index

Supplementary Table 1 1
Supplementary Table 2 2
Supplementary Table 3 2
Supplementary Figure 1 3
Supplementary Table 4 4
Supplementary Table 5 4
Supplementary Figure 2 6
Supplementary Figure 3 7
Supplementary Figure 4 8
Supplementary Figure 5 9
Supplementary Figure 6 10
Supplementary Table 6 11
Supplementary Table 7 14
Supplementary Table 8 19
Supplementary Figure 7 24
Supplementary Table 9 25
Supplementary Figure 8 26
Supplementary Figure 9 27
Supplementary Table 10 28
Supplementary Table 11 29
Supplementary Table 12 30
Supplementary Table 13 31
Supplementary Table 14 32
Supplementary Table 15 33

<u>Supplementary material</u>: 3 Results

**Country**	**Donors**	**Population**	***Per capita***	**Abs. size order**	**Rel. size order**
Cyprus	172952	1179551	14.663%	19th	1st
Israel	1185019	8713300	13.600%	5th	2nd
Germany	8505741	82685827	10.287%	2nd	3rd
Poland	1678393	37974826	4.420%	4th	4th
**Portugal**	**396545**	**10300300**	**3.850%**	**12th**	**5th**
United States	9149067	325147121	2.814%	1st	6th
Brazil	4060723	209288278	1.940%	3rd	7th
Luxembourg	11116	596336	1.864%	45th	8th
Singapore	103368	5612253	1.842%	27th	9th
Netherlands	286439	17131296	1.672%	16th	10th
Switzerland	133783	8450851	1.583%	23rd	11th
Greece	154774	10753531	1.439%	22nd	12th
Austria	123397	8797566	1.403%	25th	13th
Canada	494351	36708083	1.347%	9th	14th
Croatia	53121	4124531	1.288%	32nd	15th
Sweden	129498	10057698	1.288%	24th	16th
United Kingdom	731838	66023290	1.108%	7th	17th
Czechia	113586	10594438	1.072%	26th	18th
Denmark	59928	5764980	1.040%	31st	19th
Armenia	29980	2930450	1.023%	37th	20th
Slovenia	18690	2066388	0.904%	39th	21st
Finland	48768	5508214	0.885%	33rd	22nd
Spain	384257	46593171	0.825%	13th	23rd
Belgium	83317	11382393	0.732%	28th	24th
Italy	430635	60536709	0.711%	11th	25th
Australia	167989	24601860	0.683%	20th	26th
South Korea	344603	51466201	0.670%	14th	27th
Norway	35020	5276968	0.664%	36th	28th
Ireland	21637	4811321	0.450%	38th	29th
France	295437	67105513	0.440%	15th	30th
Lithuania	11637	2828403	0.411%	44th	31st
Japan	503795	126785797	0.397%	8th	32nd
Argentina	161248	44271041	0.364%	21st	33rd
Turkey	267330	80745020	0.331%	17th	34th
Thailand	212491	69037513	0.308%	18th	35th
Slovakia	15036	5439232	0.276%	41st	36th
New Zealand	12494	4793900	0.261%	43rd	37th
Saudi Arabia	69072	32938213	0.210%	30th	38th
Romania	36582	19583986	0.187%	34th	39th
South Africa	72843	56717156	0.128%	29th	40th
Chile	18265	18054726	0.101%	40th	41st
China	1095202	1394409267	0.079%	6th	42nd
North Macedonia	1554	2083160	0.075%	49th	43rd
Hungary	7132	9787966	0.073%	47th	44th
Serbia	3425	7020858	0.049%	48th	45th
Iran	35603	81162788	0.044%	35th	46th
India	470555	1339180127	0.035%	10th	47th
Uruguay	1065	3456750	0.031%	51st	48th
Bulgaria	1411	7075947	0.020%	50th	49th
Mexico	14060	129163276	0.011%	42nd	50th
Russia	7922	144496740	0.005%	46th	51st
Nigeria	775	190886311	0.000%	52nd	52nd

Supplementary Table 1. Absolute and relative (*per capita*) sizes of the bone marrow donor registries in 2019 according to the WMDA, correcting CEDACE's donor population to the one obtained in August 2017. Countrywide populations pertaining to 2017 extracted from the World Bank. Abs. – absolute; Rel. – relative.

<u>Supplementary material</u>: 3.1.1 Results/Descriptive analysis/Global Description

**Ancestry**	**Number of donors**	**Relative frequency**
Western	299446	99.116%
African	1984	0.657%
Mixed	535	0.177%
South Asian	88	0.029%
Eastern	50	0.017%
Hindu	13	0.004%

Supplementary Table 2. Number of donors and relative frequency according to self-declared ethnicity/ancestry.

**NUTS II Region**	**Absolute**	***Per capita***
Center	24.087%	4.103%
MA of Lisbon	28.246%	3.969%
North	35.071%	3.769%
Alentejo	6.396%	3.349%
Algarve	2.610%	2.294%
AR of Madeira	1.289%	1.909%
AR of Azores	1.033%	1.661%
Missing	1.268%	

**District**	**Absolute**	***Per capita***
Coimbra	5.215%	4.808%
Portalegre	1.324%	4.430%
Aveiro	7.921%	4.398%
Leiria	4.987%	4.200%
Lisboa	23.442%	4.131%
Braga	8.512%	3.980%
Porto	17.374%	3.791%
Santarém	4.275%	3.737%
Viseu	3.459%	3.632%
Setúbal	7.593%	3.537%
Évora	1.422%	3.382%
Guarda	1.353%	3.334%
Viana do Castelo	2.006%	3.249%
Vila Real	1.677%	3.219%
Bragança	1.010%	2.939%
Beja	0.983%	2.553%
Castelo Branco	1.247%	2.519%
Faro	2.610%	2.294%
Madeira	1.289%	1.909%
Açores	1.033%	1.661%
Missing	1.268%	

Supplementary Table 3. Absolute and *per capita* contribution of donors to CEDACE per NUTS II Region and district, sorted by *per capita* contribution.

<u>Supplementary material</u>: 3.2.1 Results/HLA frequency analysis/Global description

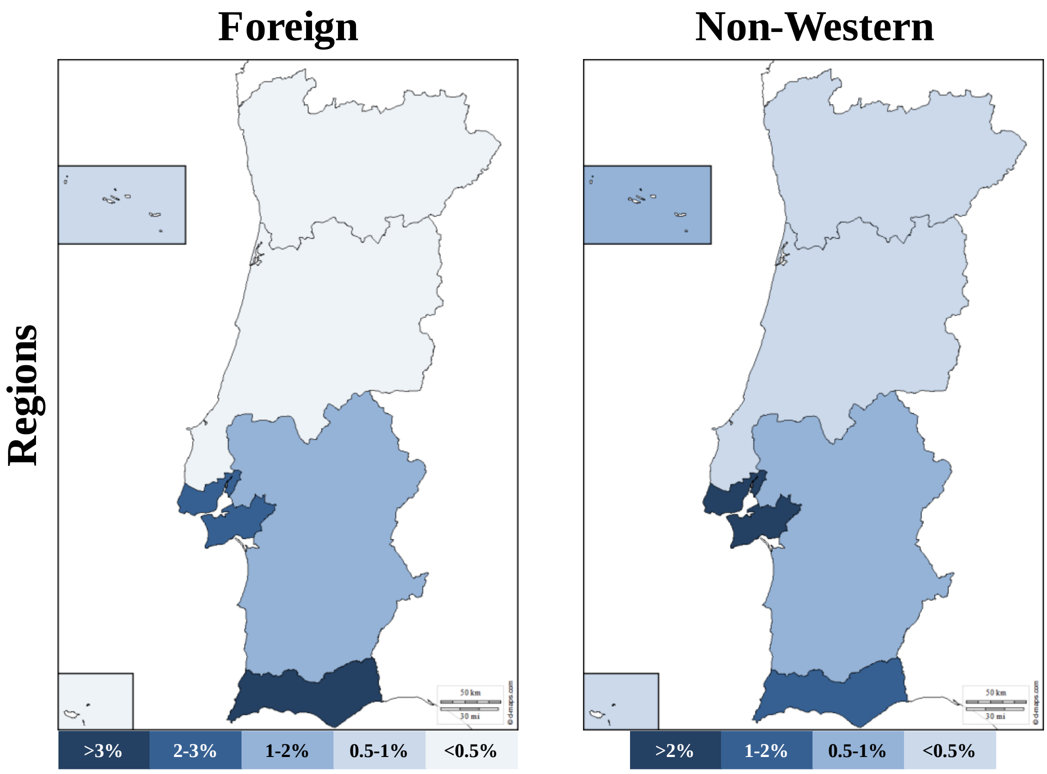

Supplementary Figure 1. Relative contribution of foreign (left) and of self-declared non-western ancestry (right) donors to the total amount of donors per NUTS II Region. Maps adapted from http://d-maps.com.

Supplementary material: 3.2.1 Results/HLA frequency analysis/Global description

NUTS II Region	L3R	L4R	L5R
North	138710	80555	2080
Metropolitan Area of Lisbon	111539	45399	1377
Center	94738	29896	1061
Alentejo	25274	10180	312
Algarve	10316	5425	115
Autonomous Region of Madeira	5106	952	68
Autonomous Region of Azores	4079	1332	53
Missing	4859	389	18

Supplementary Table 4. Number of donors per NUTS II Region in each of the datasets (**L3R**, **L4R** and **L5R**).

District	L3D
Lisbon	92567
Porto	68743
Braga	33639
Aveiro	31158
Setúbal	30000
Coimbra	20524
Leiria	19599
Santarém	16889
Viseu	13605
Faro	10316
Viana do Castelo	7938
Vila Real	6623
Évora	5603
Guarda	5307
Portalegre	5239
Madeira	5106
Castelo Branco	4934
Açores	4079
Bragança	4001
Beja	3892
Missing	4859

Supplementary Table 5. Number of donors per district in the corresponding three *loci*, low-resolution dataset (**L3D**).

<u>Supplementary material</u>: 3.2.2.1 Results/HLA frequency analysis/Allele frequencies/ Distribution of allele frequencies by NUTS II Region

HLA-A*02 was the most frequent HLA-A allele in all regions, followed by HLA-A*01 in all except the Algarve, where it was the third most frequent allele, with a frequency of 9.70%, behind HLA-A*03, with a frequency of 10.09%. HLA-A*03 was the third most frequent allele in the North, the Center, and the Alentejo; in contrast, HLA-A*24 was the third most frequent allele in the Metropolitan Area of Lisbon and in the Autonomous Regions of Madeira and Azores. The Autonomous Region of Madeira had the most disparate HLA-A allele distribution, compared to the rest of the country, with a higher frequency of HLA-A*29, HLA-A*26 and, particularly, HLA-A*33, an allele frequently identified in African and Asian populations, with a frequency more than 1.5-fold of the CEDACE registry's (5.45% vs. 3.43%, respectively).

HLA-B had a greater apparent variance in distribution across regions, although HLA-B*44 was uniformly the most frequently identified HLA-B allele. Certain alleles, such as HLA-B*51, HLA-B*35 and, more strikingly, HLA-B*18, had a clear geographically related frequency distribution, with the first having a higher frequency in the Northernmost regions and the latter two increasing in frequency towards the south of the country; all three had lower frequencies in the Autonomous Regions.

The frequency distribution of high-frequency HLA-C alleles was homogeneous across NUTS II Regions, with HLA-C*07 and HLA-C*04 appearing as the first and second most frequently identified alleles in all regions, respectively. HLA-C*06 was the third most frequently identified HLA-C allele in all regions except the Algarve, where the frequency of HLA-C*05 exceeded it.

HLA-DRB1 alleles had a frequency distribution that, as observed with some HLA-B alleles, varied according to geography. Of note, HLA-DRB1*07, HLA-DRB1*15 and HLA-DRB1*08 were more frequent in the Northern Regions, whereas HLA-DRB1*13, HLA-DRB1*03, HLA-DRB1*11 and HLA-DRB1*16 were more frequent in the Southern Regions.

HLA-DQB1*03 was the most frequently identified allele in all regions, except the Algarve, where HLA-DQB1*02 was more frequent, and the Autonomous Region of Madeira, where it was supplanted by HLA-DQB1*06. HLA-DQB1*04, the less frequently identified HLA-DQB1 allele, was most frequently identified in the Autonomous Region of Azores, where it was 4.5 times more frequent than in the Autonomous Region of Madeira.

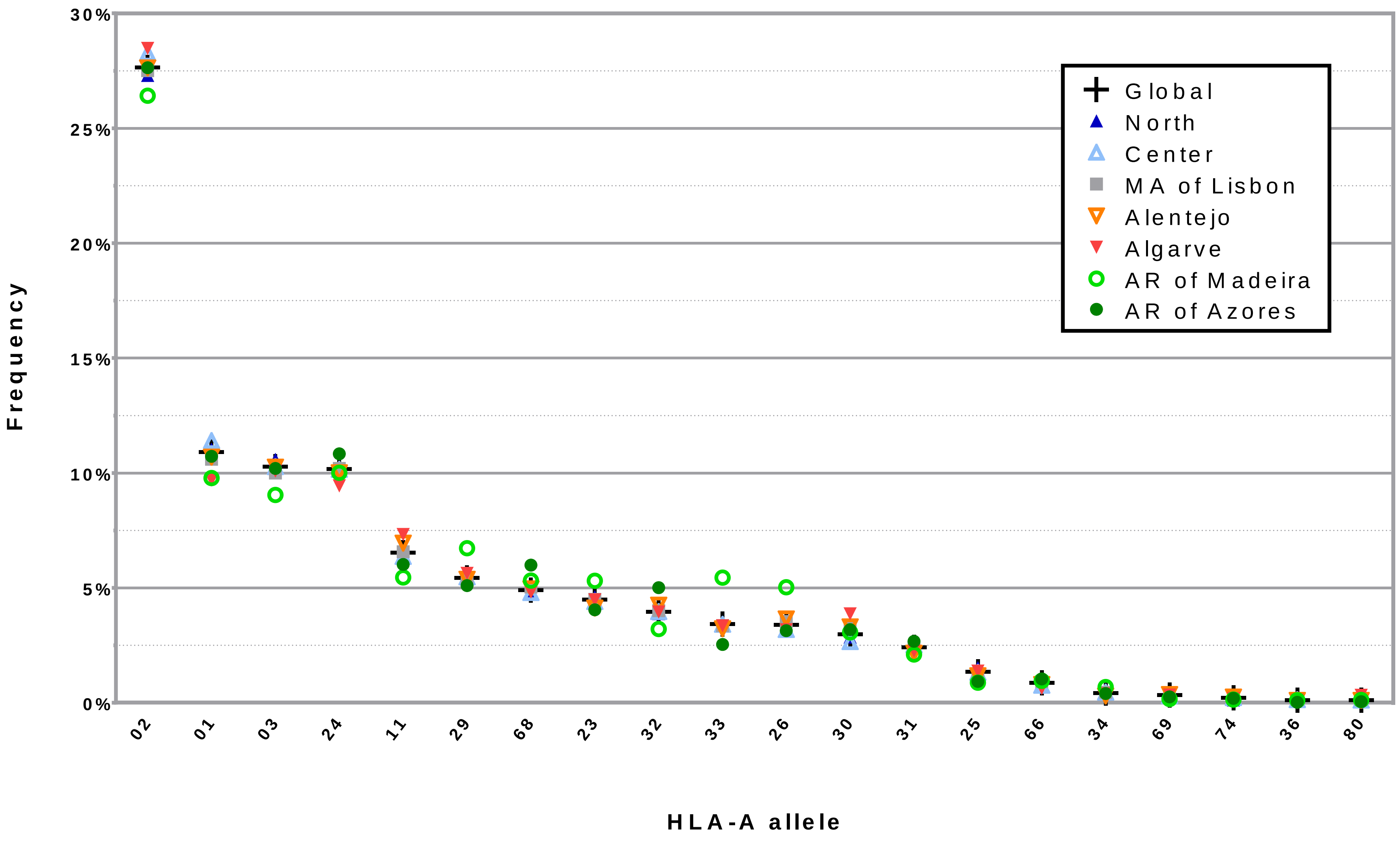

Supplementary Figure 2. Frequency distribution of low-resolution HLA-A alleles with a global frequency higher than 0.1% according to NUTS II Region. Dataset: **L3R**.

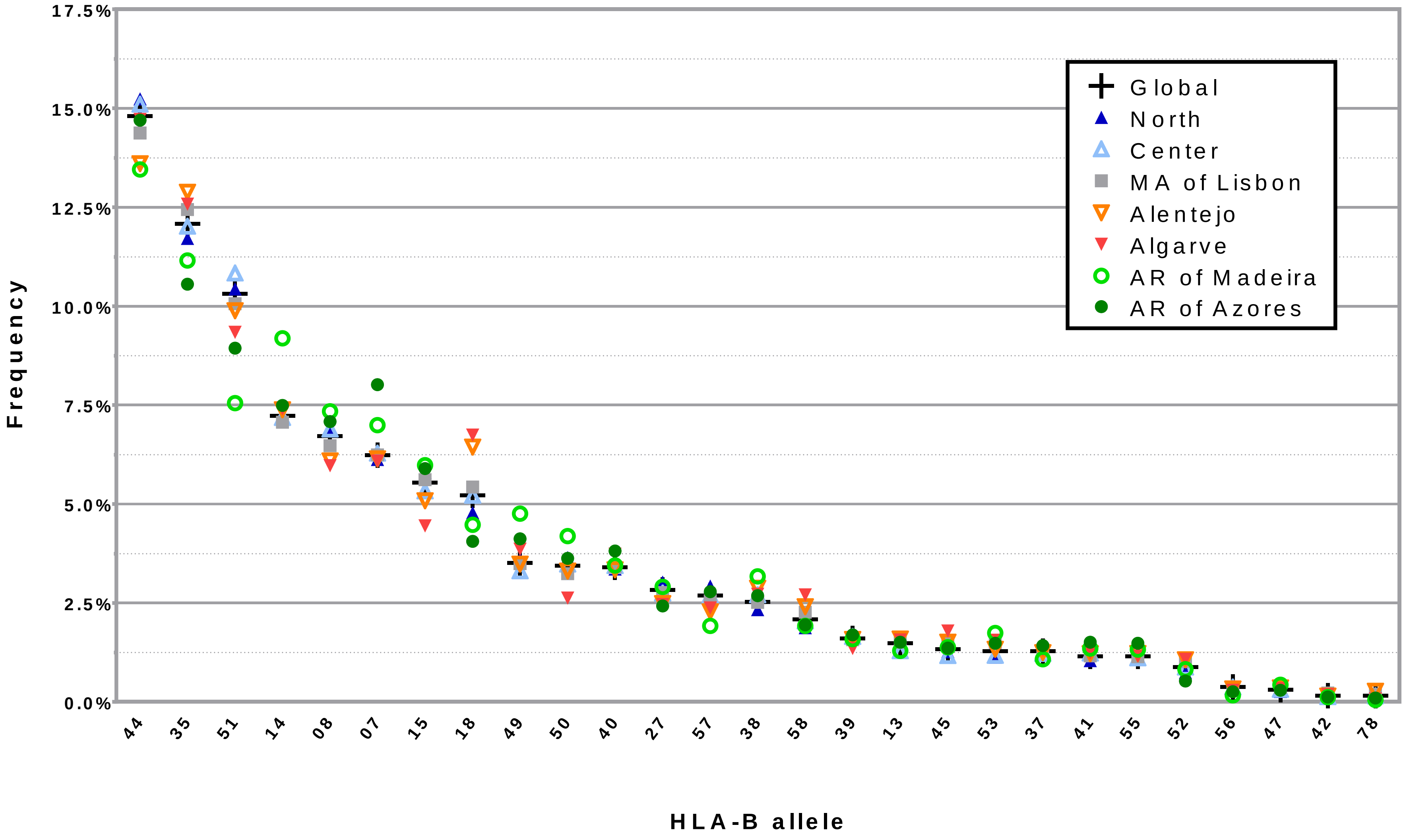

Supplementary Figure 3. Frequency distribution of low-resolution HLA-B alleles with a global frequency higher than 0.1% according to NUTS II Region. Dataset: **L3R**.

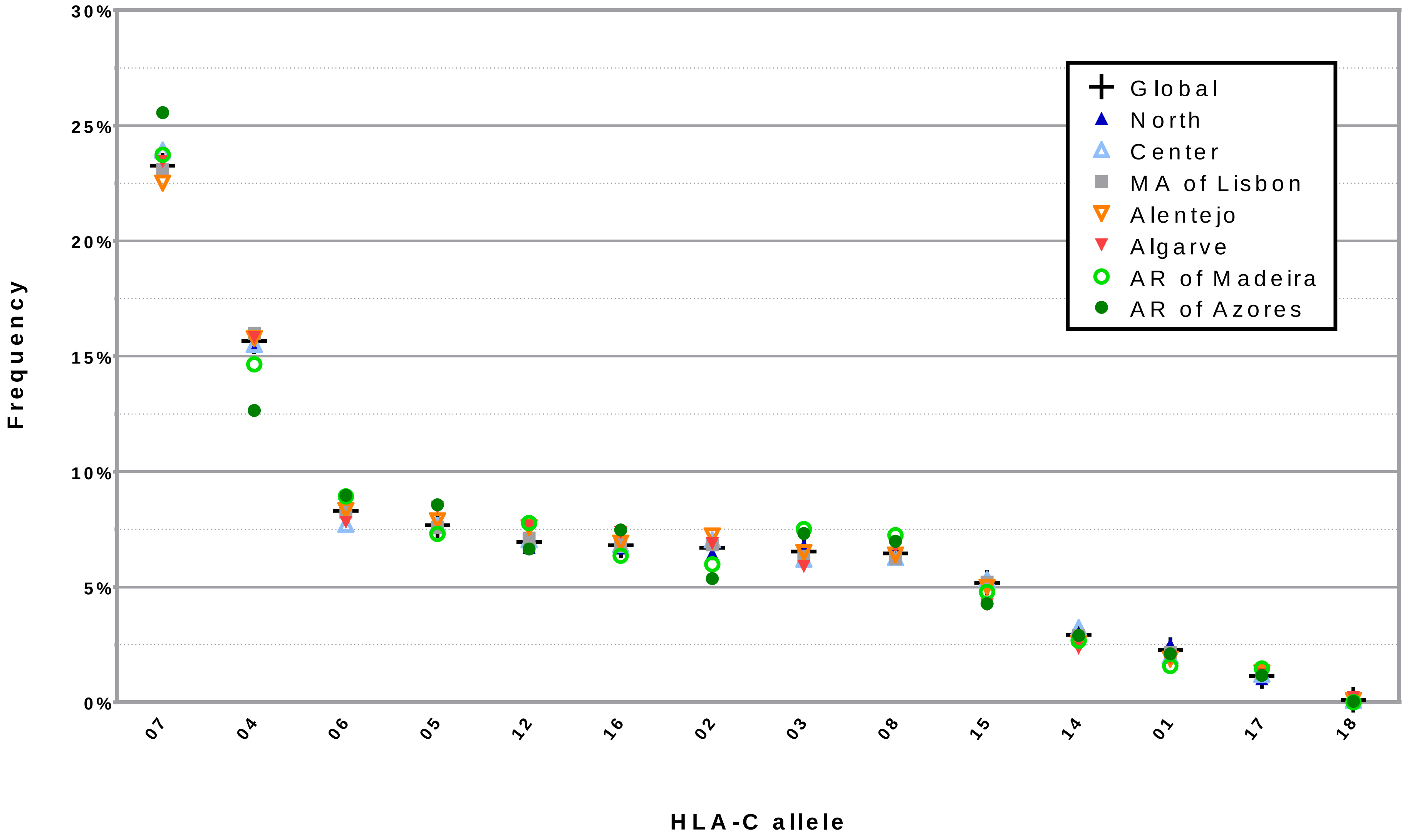

Supplementary Figure 4. Frequency distribution of low-resolution HLA-C alleles according to NUTS II Region. Dataset: **L4R**.

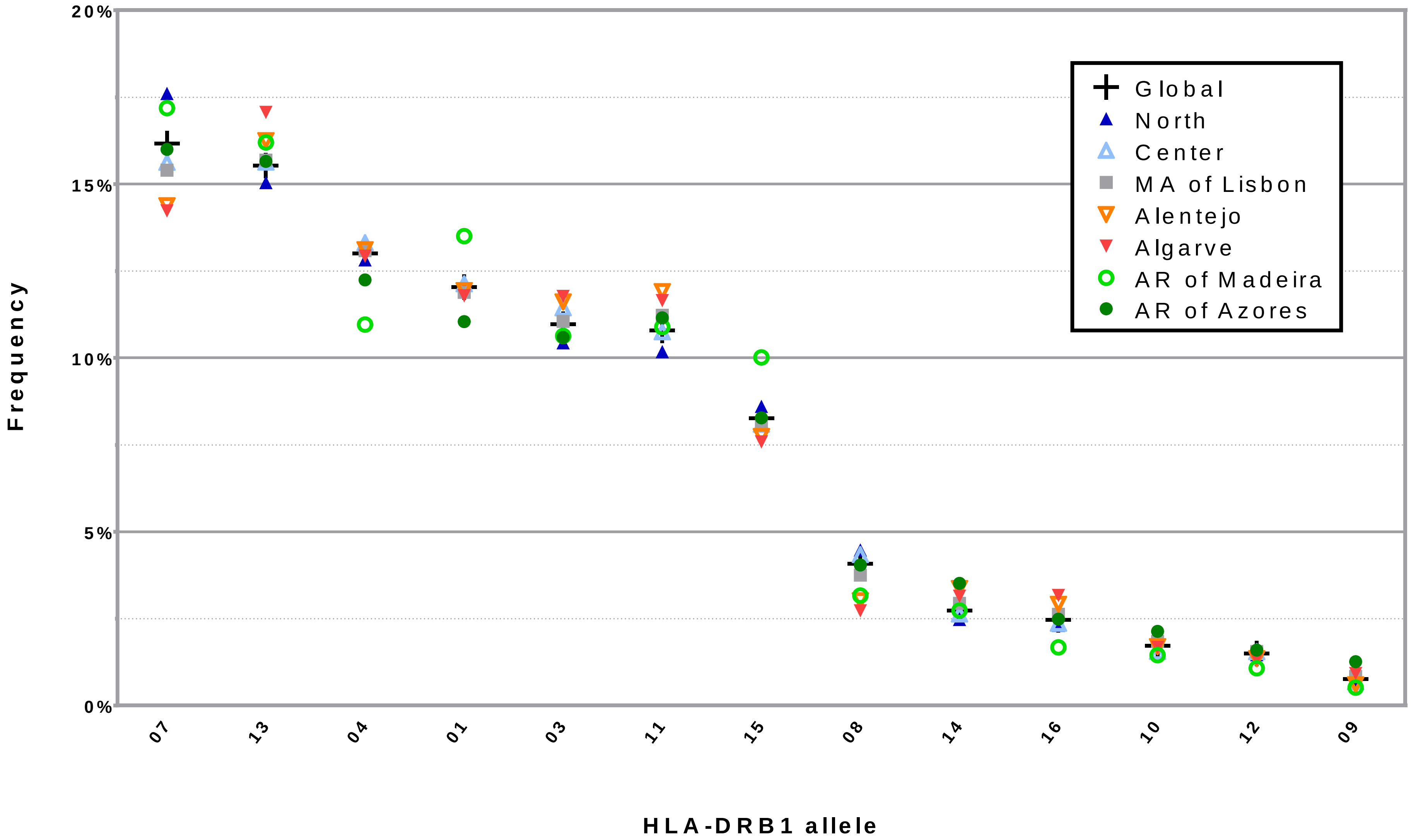

Supplementary Figure 5. Frequency distribution of low-resolution HLA-DRB1 alleles according to NUTS II Region. Dataset: **L3R**.

<u>Supplementary material</u>: 3.2.2.1 Results/HLA frequency analysis/Allele frequencies/Distribution of allele frequencies by NUTS II Region

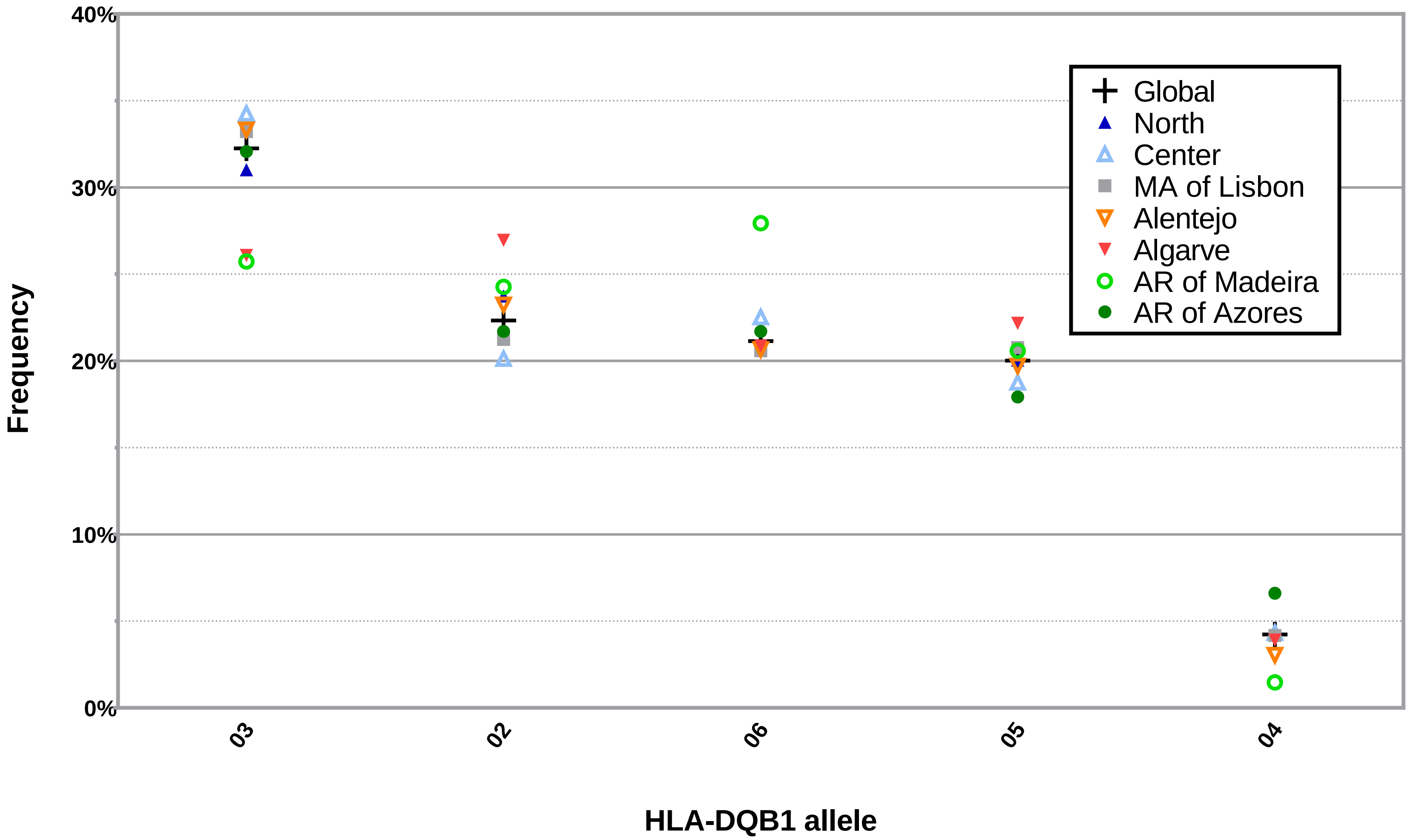

Supplementary Figure 6. Frequency distribution of low-resolution HLA-DQB1 alleles according to NUTS II Region. Dataset: **L5R**.

<u>Supplementary material</u>: 3.2.3.1.1 Results/HLA frequency analysis/Haplotype frequencies/ In the CEDACE registry, NUTS II Regions and districts/Three *loci,* low-resolution haplotype frequencies

Haplotype	Global	North	Center	MA of Lisbon	Alentejo	Algarve	AR of Madeira	AR of Azores
01 08 03	2.9784%	3.1915%	3.0198%	2.7600%	2.7580%	2.6817%	2.9215%	3.0809%
29 44 07	1.9949%	2.0498%	2.0288%	1.9364%	1.8353%	1.9205%	2.2208%	2.0723%
02 44 04	1.4742%	1.3747%	1.5814%	1.5210%	1.4063%	1.6628%	1.1249%	1.3400%
33 14 01	1.2262%	1.3661%	1.1523%	1.1137%	1.1465%	1.0983%	2.3724%	0.8052%
03 07 15	1.1802%	1.2516%	1.2260%	1.0774%	1.1074%	1.0216%	1.2351%	1.2562%
02 44 07	1.0981%	1.2090%	1.0764%	1.0239%	0.9708%	1.2718%	1.2339%	0.9314%
03 35 01	1.0120%	0.9913%	1.0575%	1.0122%	1.0831%	1.2195%	0.3593%	0.5214%
02 44 13	0.9824%	1.0648%	0.9136%	0.9012%	1.0243%	1.3059%	1.2143%	1.1395%
23 44 07	0.9495%	1.1349%	0.9407%	0.8156%	0.7158%	0.6602%	0.9765%	0.7472%
02 51 08	0.9191%	0.8190%	1.1900%	0.9048%	0.6996%	0.4614%	0.6075%	0.9241%
11 35 01	0.9164%	0.9050%	0.8871%	0.9372%	0.8266%	1.1746%	1.0562%	1.1268%
02 50 07	0.8603%	0.9712%	0.8781%	0.7444%	0.8121%	0.4353%	1.2959%	0.8069%
02 51 11	0.8598%	0.7498%	0.9851%	0.8896%	0.9372%	0.9206%	0.4938%	0.7365%
02 51 13	0.7574%	0.7770%	0.7515%	0.7017%	0.8200%	0.9407%	0.8199%	0.6261%
02 07 15	0.6833%	0.6998%	0.7134%	0.6638%	0.5519%	0.5827%	0.9719%	1.0450%
24 35 11	0.6431%	0.5195%	0.6287%	0.7647%	0.7472%	0.6824%	0.8426%	0.7882%
02 18 11	0.6248%	0.5028%	0.6014%	0.6877%	0.9669%	0.8032%	1.1152%	0.4209%
01 57 07	0.6179%	0.7525%	0.5052%	0.5773%	0.6162%	0.5467%	0.3629%	0.8545%
02 14 01	0.5944%	0.5591%	0.6264%	0.5724%	0.6679%	0.8406%	0.6217%	0.6728%
02 51 07	0.5538%	0.4850%	0.6693%	0.5562%	0.5768%	0.4527%	0.2724%	0.4538%
30 18 03	0.5488%	0.4391%	0.5427%	0.6166%	0.7723%	1.0511%	0.3034%	0.4539%
02 08 03	0.5135%	0.5830%	0.4792%	0.4905%	0.4093%	0.5619%	0.4331%	0.4518%
02 18 03	0.5104%	0.3564%	0.5423%	0.6078%	0.6974%	0.9037%	0.1393%	0.5525%
02 35 11	0.4992%	0.5620%	0.4583%	0.4556%	0.4426%	0.4249%	0.9684%	0.3819%
02 51 04	0.4970%	0.4617%	0.5660%	0.4950%	0.5549%	0.6185%	0.3369%	0.1885%
02 15 04	0.4712%	0.4721%	0.4990%	0.4701%	0.4028%	0.3978%	0.5533%	0.6192%
24 14 01	0.4243%	0.4726%	0.4457%	0.3671%	0.3144%	0.4603%	0.6370%	0.2978%
02 35 01	0.4226%	0.4494%	0.4034%	0.4078%	0.4533%	0.4358%	0.4691%	0.4458%
24 07 15	0.4159%	0.4394%	0.4077%	0.3904%	0.3984%	0.3583%	0.4995%	0.4386%
26 38 13	0.4050%	0.3483%	0.4342%	0.4081%	0.5196%	0.3462%	0.9065%	0.4950%
24 08 03	0.4021%	0.4582%	0.3818%	0.3606%	0.3303%	0.3852%	0.5170%	0.4326%
03 14 01	0.3998%	0.3778%	0.4253%	0.3928%	0.4369%	0.5468%	0.3787%	0.3265%
02 35 03	0.3962%	0.3360%	0.4934%	0.4036%	0.4210%	0.2482%	0.2531%	0.5214%
02 13 07	0.3646%	0.3433%	0.3538%	0.3909%	0.4125%	0.4665%	0.3991%	0.2358%
02 44 11	0.3624%	0.4023%	0.2872%	0.3716%	0.3474%	0.3107%	0.3615%	0.4182%
30 13 07	0.3550%	0.4256%	0.2472%	0.3555%	0.4097%	0.4519%	0.0828%	0.4071%
03 35 11	0.3532%	0.3081%	0.4067%	0.3629%	0.4158%	0.3330%	0.2519%	0.2738%
24 15 13	0.3493%	0.3854%	0.3221%	0.3057%	0.4513%	0.2754%	0.4832%	0.4626%
25 18 15	0.3393%	0.3702%	0.3480%	0.3235%	0.2527%	0.3473%	0.3113%	0.1637%
68 53 13	0.3129%	0.2423%	0.3352%	0.3536%	0.3920%	0.3089%	0.1822%	0.6325%
02 57 07	0.2898%	0.3020%	0.3366%	0.2687%	0.2411%	0.1926%	0.1133%	0.3399%
02 44 15	0.2767%	0.2979%	0.2897%	0.2542%	0.2363%	0.1945%	0.1592%	0.4331%
03 35 13	0.2691%	0.3153%	0.2242%	0.2696%	0.2072%	0.2217%	0.2506%	0.2349%
32 14 07	0.2688%	0.3066%	0.2259%	0.2790%	0.2615%	0.1579%	0.1028%	0.3050%
31 40 04	0.2687%	0.2357%	0.3283%	0.2556%	0.2460%	0.2775%	0.3783%	0.3482%
02 51 15	0.2680%	0.2962%	0.2308%	0.2705%	0.2849%	0.2441%	0.1320%	0.2157%
02 35 13	0.2665%	0.2503%	0.2522%	0.2663%	0.2856%	0.4219%	0.3170%	0.3739%
02 15 13	0.2633%	0.2673%	0.2426%	0.2626%	0.3048%	0.3034%	0.3952%	0.3373%
24 38 13	0.2627%	0.2026%	0.3057%	0.2587%	0.3880%	0.3159%	0.3862%	0.3703%
11 35 11	0.2614%	0.2246%	0.2619%	0.2710%	0.4435%	0.2842%	0.3827%	0.1260%
02 49 04	0.2613%	0.3397%	0.2018%	0.2315%	0.2278%	0.2289%	0.2271%	0.1989%
02 44 01	0.2553%	0.2777%	0.2522%	0.2401%	0.2436%	0.1617%	0.2550%	0.2881%
02 44 16	0.2509%	0.2193%	0.2313%	0.3007%	0.3143%	0.4571%	0.0841%	0.1293%
34 08 03	0.2398%	0.2248%	0.2966%	0.2146%	0.1794%	0.1874%	0.4185%	0.2240%
33 14 03	0.2350%	0.1813%	0.2983%	0.2261%	0.2598%	0.2902%	0.5400%	0.1847%
24 35 04	0.2347%	0.1962%	0.2447%	0.2725%	0.2755%	0.1949%	0.1248%	0.1048%

Supplementary material: 3.2.3.1.1 Results/HLA frequency analysis/Haplotype frequencies/ In the CEDACE registry, NUTS II Regions and districts/Three *loci*, low-resolution haplotype frequencies

Haplotype	Global	North	Center	MA of Lisbon	Alentejo	Algarve	AR of Madeira	AR of Azores
03 51 11	0.2341%	0.2599%	0.2389%	0.2088%	0.1798%	0.3083%	0.1438%	0.2130%
01 52 15	0.2332%	0.1967%	0.2380%	0.2690%	0.2607%	0.2475%	0.3036%	0.1425%
01 37 10	0.2327%	0.2306%	0.2471%	0.2276%	0.2509%	0.2090%	0.0867%	0.3063%
01 51 04	0.2298%	0.2677%	0.2463%	0.1915%	0.1633%	0.2065%	0.1296%	0.1296%
24 44 13	0.2273%	0.2447%	0.2390%	0.1982%	0.1984%	0.2743%	0.1901%	0.3148%
01 35 04	0.2176%	0.2390%	0.2018%	0.2076%	0.2687%	0.2040%	0.0907%	0.0895%
01 44 07	0.2172%	0.2399%	0.2392%	0.2063%	0.1452%	0.0745%	0.0692%	0.1729%
24 35 07	0.2162%	0.2196%	0.2348%	0.2072%	0.1871%	0.0653%	0.2716%	0.2194%
24 44 07	0.2153%	0.2333%	0.2064%	0.2114%	0.1890%	0.2300%	0.1323%	0.1695%
02 15 01	0.2140%	0.2079%	0.2477%	0.2117%	0.1411%	0.2116%	0.0668%	0.2001%
26 38 04	0.2136%	0.2081%	0.2240%	0.2144%	0.2072%	0.3153%	0.1692%	0.2227%
24 15 11	0.2133%	0.2294%	0.2168%	0.1889%	0.1954%	0.1611%	0.3233%	0.2628%
68 51 13	0.2122%	0.2289%	0.1748%	0.2207%	0.2135%	0.1175%	0.5028%	0.1658%
02 40 13	0.2113%	0.1759%	0.2264%	0.2583%	0.1993%	0.2119%	0.1482%	0.3083%
02 44 12	0.2074%	0.2636%	0.1936%	0.1868%	0.1470%	0.1015%	0.0735%	0.0950%
02 50 04	0.2072%	0.1972%	0.2707%	0.1616%	0.2412%	0.1866%	0.1805%	0.1599%
03 18 13	0.2056%	0.3416%	0.1394%	0.1196%	0.1824%	0.0779%	0.0674%	0.2488%
01 08 07	0.2044%	0.2653%	0.1977%	0.1594%	0.1268%	0.1219%	0.2473%	0.0884%
23 49 13	0.2041%	0.2360%	0.1658%	0.2033%	0.2205%	0.2182%	0.1487%	0.2100%
32 40 13	0.2033%	0.1548%	0.2413%	0.1881%	0.3011%	0.4565%	0.2872%	0.0597%
01 15 13	0.2026%	0.2238%	0.2163%	0.1958%	0.1206%	0.1652%	0.0838%	0.1244%
24 35 13	0.2021%	0.2045%	0.1528%	0.2316%	0.2212%	0.4514%	0.2890%	0.0033%
02 50 13	0.1983%	0.1554%	0.2889%	0.1821%	0.2290%	0.1557%	0.0777%	0.0531%
02 39 16	0.1961%	0.1629%	0.2119%	0.2103%	0.2828%	0.2147%	0.1431%	0.2361%
02 15 11	0.1959%	0.1781%	0.2276%	0.2147%	0.2020%	0.1043%	0.0829%	0.1687%
02 58 07	0.1935%	0.1720%	0.2354%	0.1956%	0.1956%	0.0828%	0.2303%	0.1158%
02 51 01	0.1934%	0.2282%	0.1548%	0.1896%	0.1599%	0.1776%	0.2617%	0.2860%
31 51 13	0.1927%	0.1944%	0.2160%	0.1803%	0.1677%	0.2670%	0.0149%	0.1655%
11 35 14	0.1909%	0.1796%	0.2015%	0.1880%	0.2314%	0.2067%	0.0821%	0.0238%
24 35 14	0.1902%	0.1548%	0.1594%	0.2525%	0.2582%	0.2087%	0.0089%	0.3131%
03 08 03	0.1896%	0.1813%	0.1989%	0.1921%	0.1880%	0.1162%	0.2939%	0.2679%
11 49 04	0.1874%	0.1802%	0.1578%	0.2093%	0.1759%	0.3698%	0.1298%	0.0632%
29 44 13	0.1856%	0.1911%	0.1610%	0.1896%	0.1878%	0.1424%	0.4637%	0.2694%
03 07 13	0.1849%	0.1343%	0.2330%	0.1954%	0.2276%	0.0998%	0.0634%	0.2591%
32 35 04	0.1843%	0.2269%	0.1390%	0.1688%	0.2777%	0.1352%	0.0411%	0.0564%
02 49 11	0.1816%	0.0969%	0.2806%	0.1741%	0.2247%	0.1507%	0.3463%	0.4712%
01 08 13	0.1813%	0.1775%	0.2117%	0.1783%	0.1692%	0.0795%	0.0707%	0.2012%
02 27 01	0.1796%	0.2073%	0.1709%	0.1668%	0.1701%	0.1099%	0.1694%	0.0832%
33 44 01	0.1792%	0.1621%	0.2516%	0.1584%	0.1250%	0.1968%	0.0473%	0.0967%
01 44 04	0.1770%	0.1904%	0.2005%	0.1439%	0.2298%	0.0650%	0.0041%	0.2776%
24 35 01	0.1770%	0.1834%	0.1805%	0.1725%	0.1391%	0.3339%	0.1470%	0.2711%
33 14 13	0.1766%	0.2081%	0.1439%	0.1667%	0.2064%	0.1173%	0.2096%	0.1924%
31 51 04	0.1759%	0.1791%	0.1681%	0.1830%	0.2002%	0.1115%	0.1064%	0.0530%
29 44 04	0.1756%	0.2059%	0.1550%	0.1687%	0.1749%	0.1440%	0.0775%	0.0000%
02 07 01	0.1753%	0.1091%	0.2461%	0.1688%	0.1638%	0.1839%	0.4355%	0.4423%
29 15 13	0.1747%	0.1328%	0.1694%	0.2082%	0.2419%	0.1229%	0.4853%	0.1455%
23 44 01	0.1737%	0.1666%	0.1987%	0.1734%	0.1310%	0.0621%	0.3281%	0.1360%
23 44 13	0.1733%	0.1951%	0.1853%	0.1578%	0.1422%	0.1019%	0.0650%	0.1001%
24 51 11	0.1726%	0.1830%	0.1919%	0.1740%	0.1464%	0.0966%	0.1025%	0.0000%
03 07 04	0.1718%	0.1743%	0.1745%	0.1760%	0.1422%	0.1020%	0.1185%	0.1387%
68 15 15	0.1718%	0.1616%	0.2279%	0.1606%	0.1213%	0.0743%	0.0682%	0.0000%
02 41 13	0.1715%	0.1710%	0.1556%	0.1740%	0.2007%	0.3080%	0.0807%	0.1215%
02 35 14	0.1710%	0.1782%	0.1563%	0.1791%	0.1890%	0.0568%	0.2154%	0.1707%
02 51 14	0.1673%	0.1109%	0.1925%	0.1968%	0.2318%	0.1869%	0.1782%	0.1664%
24 45 10	0.1663%	0.1521%	0.1573%	0.1868%	0.1883%	0.1800%	0.0587%	0.2795%
01 08 11	0.1660%	0.1319%	0.1941%	0.1601%	0.2027%	0.2033%	0.3843%	0.1878%

Supplementary material: 3.2.3.1.1 Results/HLA frequency analysis/Haplotype frequencies/ In the CEDACE registry, NUTS II Regions and districts/Three *loci*, low-resolution haplotype frequencies

Haplotype	Global	North	Center	MA of Lisbon	Alentejo	Algarve	AR of Madeira	AR of Azores
02 15 07	0.1655%	0.2293%	0.1214%	0.1390%	0.1437%	0.1277%	0.1045%	0.0481%
11 44 13	0.1637%	0.1048%	0.2259%	0.1753%	0.1378%	0.1502%	0.3153%	0.2779%
02 44 08	0.1626%	0.1978%	0.1529%	0.1545%	0.0937%	0.1356%	0.0765%	0.1408%
24 27 04	0.1617%	0.1358%	0.2064%	0.1642%	0.1712%	0.1183%	0.0632%	0.1379%
33 14 07	0.1614%	0.2019%	0.1258%	0.1377%	0.1325%	0.0848%	0.5118%	0.2246%
02 51 16	0.1613%	0.1486%	0.1271%	0.1833%	0.1572%	0.3817%	0.0450%	0.3571%
24 50 04	0.1607%	0.1451%	0.1982%	0.1793%	0.1038%	0.1248%	0.0000%	0.0735%
11 35 13	0.1599%	0.1102%	0.2100%	0.1807%	0.2007%	0.2346%	0.0000%	0.0000%
03 35 15	0.1581%	0.2175%	0.1048%	0.1242%	0.1744%	0.2409%	0.2172%	0.0256%
03 07 07	0.1580%	0.1837%	0.1467%	0.1492%	0.1394%	0.1613%	0.1982%	0.0426%
02 14 03	0.1572%	0.0989%	0.1984%	0.1892%	0.2169%	0.1045%	0.1594%	0.1238%
02 07 13	0.1569%	0.1233%	0.2047%	0.1474%	0.2048%	0.1414%	0.1407%	0.2130%
03 51 04	0.1561%	0.1667%	0.1698%	0.1506%	0.1673%	0.0969%	0.0305%	0.0667%
02 27 13	0.1560%	0.1491%	0.1623%	0.1676%	0.1576%	0.2286%	0.0372%	0.0894%
11 14 01	0.1546%	0.2102%	0.1054%	0.1347%	0.1429%	0.1334%	0.0979%	0.2820%
02 58 13	0.1530%	0.1251%	0.1330%	0.1776%	0.2326%	0.2643%	0.0972%	0.2362%
11 35 07	0.1511%	0.1246%	0.1776%	0.1548%	0.1621%	0.1007%	0.0841%	0.3395%
03 44 07	0.1495%	0.1405%	0.1657%	0.1499%	0.1409%	0.0760%	0.1481%	0.0818%
32 49 04	0.1493%	0.1469%	0.1281%	0.1594%	0.2031%	0.1295%	0.2929%	0.0738%
11 35 04	0.1490%	0.1768%	0.1212%	0.1572%	0.1142%	0.1436%	0.0537%	0.1397%
24 35 15	0.1481%	0.1524%	0.1410%	0.1621%	0.1525%	0.0673%	0.0905%	0.1602%
02 49 01	0.1476%	0.1778%	0.1384%	0.1328%	0.1559%	0.0795%	0.0328%	0.1304%
02 14 07	0.1468%	0.0985%	0.1604%	0.1788%	0.1636%	0.2879%	0.1472%	0.2298%
11 14 07	0.1451%	0.1387%	0.1172%	0.1526%	0.1636%	0.3297%	0.2158%	0.1469%
29 44 11	0.1446%	0.1787%	0.1108%	0.1371%	0.1375%	0.0998%	0.0207%	0.2775%
23 14 01	0.1436%	0.1564%	0.1545%	0.1442%	0.0758%	0.0752%	0.0242%	0.2079%
26 44 07	0.1434%	0.1711%	0.1116%	0.1440%	0.1084%	0.1206%	0.2460%	0.1607%
01 35 11	0.1432%	0.1081%	0.2092%	0.1238%	0.1513%	0.2512%	0.0923%	0.2805%
11 27 01	0.1428%	0.1767%	0.1345%	0.1224%	0.1092%	0.0661%	0.1893%	0.0645%
02 38 13	0.1424%	0.1378%	0.1523%	0.1467%	0.1415%	0.1068%	0.0602%	0.1613%
68 51 07	0.1423%	0.1601%	0.1555%	0.1217%	0.1182%	0.0873%	0.0150%	0.0699%
01 35 13	0.1412%	0.1392%	0.1507%	0.1355%	0.1609%	0.1463%	0.0366%	0.1758%
02 50 03	0.1406%	0.1590%	0.1321%	0.1318%	0.1177%	0.0942%	0.1529%	0.2380%
03 35 07	0.1406%	0.1838%	0.0865%	0.1131%	0.1563%	0.1226%	0.5161%	0.1272%
32 14 01	0.1406%	0.1585%	0.1086%	0.1401%	0.1426%	0.0996%	0.0676%	0.2017%
02 35 04	0.1405%	0.1092%	0.1339%	0.1727%	0.1894%	0.1754%	0.1004%	0.1953%
24 51 04	0.1402%	0.1405%	0.1236%	0.1539%	0.1616%	0.1811%	0.0713%	0.0970%
02 51 03	0.1401%	0.1288%	0.1256%	0.1479%	0.2114%	0.2861%	0.0169%	0.0952%

Supplementary Table 6. Three *loci*, low-resolution frequencies of the 150 most frequent HLA-A/-B/-DRB1 haplotypes in CEDACE and corresponding frequencies according to NUTS II Region. Dataset: **L3R**.

<u>Supplementary material</u>: 3.2.3.1.1 Results/HLA frequency analysis/Haplotype frequencies/In the CEDACE registry, NUTS II Regions and districts/ Three *loci*, low-resolution haplotype frequencies

Haplotype	Viana do Castelo	Braga	Vila Real	Bragança	Porto	Aveiro	Viseu	Guarda	Coimbra	Castelo Branco	Leiria
01 08 03	2.5555%	3.3107%	3.4222%	2.7716%	3.2575%	3.1634%	2.7355%	2.8076%	3.1563%	3.5183%	2.9726%
29 44 07	2.3430%	2.3286%	2.5234%	2.2285%	1.9223%	1.7891%	1.7496%	2.1066%	2.2068%	1.1497%	2.3178%
02 44 04	1.3824%	1.1983%	0.9355%	1.2351%	1.3850%	1.9246%	1.3762%	1.3681%	1.8588%	1.5439%	1.3866%
33 14 01	1.3182%	1.4580%	1.0603%	1.5062%	1.3474%	1.1275%	1.1980%	1.0061%	1.2202%	1.3233%	1.2832%
03 07 15	1.1930%	1.2832%	1.0382%	0.9903%	1.2723%	1.2282%	1.2770%	1.3660%	1.2731%	1.1097%	1.3224%
02 44 07	0.6653%	0.9927%	0.9781%	0.9243%	1.3116%	1.4787%	1.2170%	1.2720%	1.1169%	1.0624%	0.9286%
03 35 01	0.7740%	0.9278%	0.7360%	0.8126%	1.0187%	1.3339%	0.9696%	1.2036%	0.7512%	0.9525%	1.2103%
02 44 13	1.1149%	1.2065%	0.7364%	0.7534%	1.1074%	1.0261%	0.7848%	0.8945%	0.8951%	0.8973%	0.8863%
23 44 07	1.1527%	1.2098%	1.1468%	0.9000%	0.9560%	1.5174%	1.1361%	1.1287%	0.7712%	0.3960%	0.9516%
02 51 08	1.3912%	0.8046%	0.6608%	0.7814%	0.7395%	1.4051%	1.3038%	0.9800%	1.3124%	1.3355%	0.9233%
11 35 01	0.8051%	0.8237%	0.7817%	0.8482%	0.9300%	0.9721%	0.8944%	0.8400%	0.9523%	0.8577%	0.8334%
02 50 07	0.7331%	0.8143%	0.8760%	0.6938%	1.0721%	1.2119%	0.7858%	0.6249%	0.8109%	0.5254%	0.9513%
02 51 11	0.6809%	0.6116%	0.7480%	1.1374%	0.7634%	0.8702%	0.9057%	1.1928%	1.0923%	1.4768%	0.9553%
02 51 13	1.0117%	0.9028%	0.7424%	0.6013%	0.7274%	0.7311%	0.9146%	0.7285%	0.6402%	0.9734%	0.6534%
02 07 15	0.6991%	0.8653%	0.5349%	0.3855%	0.6541%	0.7634%	0.6757%	0.5361%	0.7259%	0.5848%	0.8517%
24 35 11	0.7158%	0.5491%	0.5493%	0.5439%	0.5253%	0.3306%	0.5778%	0.8414%	0.7373%	0.9287%	0.7209%
02 18 11	0.4973%	0.4517%	0.5205%	1.0360%	0.5354%	0.4644%	0.2220%	0.5369%	0.5863%	1.0366%	0.9178%
01 57 07	0.6333%	0.8082%	0.7753%	0.7530%	0.8131%	0.4736%	0.5778%	0.3892%	0.4842%	0.3394%	0.4731%
02 14 01	0.7352%	0.5840%	0.6211%	0.9588%	0.5334%	0.6190%	0.4905%	0.5031%	0.8539%	0.7683%	0.6259%
02 51 07	0.9387%	0.6085%	0.3444%	0.2456%	0.4481%	0.4539%	0.6572%	0.5233%	0.4458%	0.6169%	0.8324%
30 18 03	0.1744%	0.3403%	0.3835%	0.5498%	0.4472%	0.6326%	0.4978%	0.6563%	0.4851%	0.5779%	0.6529%
02 08 03	0.3682%	0.6423%	0.4222%	0.4526%	0.5477%	0.7765%	0.5135%	0.4287%	0.4972%	0.2870%	0.4288%
02 18 03	0.1595%	0.3549%	0.6119%	1.0055%	0.3503%	0.2738%	0.4638%	0.3498%	0.6156%	0.4393%	0.8858%
02 35 11	0.2234%	0.3635%	0.5594%	0.4515%	0.6473%	0.6334%	0.4865%	0.8601%	0.5633%	0.6075%	0.3796%
02 51 04	0.4770%	0.4495%	0.4306%	0.5444%	0.4322%	0.5414%	0.7529%	0.5810%	0.5721%	0.5103%	0.4064%
02 15 04	0.2705%	0.5270%	0.3625%	0.3115%	0.4814%	0.5826%	0.6353%	0.3959%	0.5144%	0.5164%	0.3982%
24 14 01	0.5776%	0.5253%	0.3495%	0.0960%	0.4724%	0.4449%	0.3645%	0.3283%	0.4063%	0.4746%	0.4766%
02 35 01	0.5056%	0.3670%	0.5722%	0.6266%	0.4355%	0.5817%	0.3511%	0.3436%	0.4588%	0.3706%	0.3949%
24 07 15	0.4704%	0.4770%	0.1196%	0.5577%	0.4356%	0.4447%	0.5602%	0.2938%	0.3039%	0.3417%	0.4673%
26 38 13	0.5106%	0.4458%	0.3140%	0.4929%	0.2975%	0.4182%	0.3175%	0.3782%	0.4897%	0.6700%	0.3662%
24 08 03	0.3644%	0.3746%	0.2382%	0.1702%	0.5184%	0.5185%	0.3293%	0.2175%	0.3609%	0.4031%	0.3658%
03 14 01	0.4443%	0.4206%	0.4192%	0.4889%	0.3276%	0.4234%	0.4595%	0.4930%	0.4950%	0.4042%	0.3157%
02 35 03	0.1481%	0.4523%	0.3344%	0.3750%	0.2980%	0.3346%	0.4951%	0.2741%	0.6469%	0.5796%	0.4501%
02 13 07	0.6524%	0.4546%	0.4261%	0.3373%	0.2729%	0.1959%	0.4012%	0.1412%	0.3790%	0.1798%	0.3552%
02 44 11	0.2484%	0.4572%	0.5317%	0.4576%	0.4115%	0.2852%	0.3728%	0.5593%	0.2282%	0.6247%	0.1819%

Supplementary material: 3.2.3.1.1 Results/HLA frequency analysis/Haplotype frequencies/In the CEDACE registry, NUTS II Regions and districts/ Three *loci*, low-resolution haplotype frequencies

Haplotype	Viana do Castelo	Braga	Vila Real	Bragança	Porto	Aveiro	Viseu	Guarda	Coimbra	Castelo Branco	Leiria
30 13 07	0.7096%	0.3906%	0.4140%	0.5832%	0.4444%	0.2202%	0.2605%	0.4194%	0.2181%	0.2938%	0.2148%
03 35 11	0.1958%	0.2597%	0.2702%	0.3720%	0.3121%	0.4864%	0.4699%	0.5331%	0.2594%	0.2535%	0.2574%
24 15 13	0.5535%	0.4824%	0.3981%	0.3259%	0.3593%	0.3079%	0.2738%	0.5416%	0.2951%	0.2725%	0.4017%
25 18 15	0.4359%	0.3932%	0.3434%	0.2632%	0.3594%	0.3679%	0.2592%	0.3885%	0.2585%	0.3151%	0.4185%
68 53 13	0.4223%	0.2499%	0.1459%	0.4453%	0.2458%	0.1859%	0.2875%	0.3815%	0.2975%	0.3464%	0.4362%
02 57 07	0.2466%	0.3722%	0.5175%	0.2172%	0.2817%	0.3210%	0.4915%	0.3141%	0.3129%	0.3167%	0.2366%
02 44 15	0.4339%	0.2649%	0.3005%	0.3163%	0.2606%	0.3834%	0.3019%	0.3463%	0.2822%	0.2872%	0.2723%
03 35 13	0.2721%	0.3372%	0.2955%	0.4653%	0.3226%	0.2220%	0.3071%	0.4129%	0.1870%	0.1778%	0.2346%
32 14 07	0.6022%	0.3024%	0.3765%	0.1995%	0.2905%	0.1893%	0.3497%	0.3470%	0.2382%	0.1751%	0.1851%
31 40 04	0.2683%	0.2322%	0.1914%	0.0975%	0.2623%	0.2440%	0.1214%	0.1110%	0.5131%	0.1442%	0.3536%
02 51 15	0.3585%	0.4136%	0.3483%	0.3436%	0.2328%	0.2891%	0.2577%	0.2998%	0.1786%	0.1544%	0.1733%
02 35 13	0.2449%	0.2362%	0.6309%	0.2345%	0.2538%	0.1923%	0.3112%	0.2922%	0.1815%	0.3598%	0.2491%
02 15 13	0.3244%	0.1858%	0.1450%	0.6753%	0.3290%	0.1801%	0.2579%	0.5167%	0.2901%	0.2068%	0.2606%
24 38 13	0.0827%	0.1329%	0.1623%	0.1070%	0.2789%	0.2752%	0.2354%	0.3598%	0.3563%	0.4461%	0.2226%
11 35 11	0.0597%	0.1425%	0.1406%	0.1895%	0.2868%	0.3136%	0.1980%	0.1247%	0.3791%	0.2675%	0.2473%
02 49 04	0.1045%	0.3405%	0.4465%	0.3293%	0.3801%	0.2366%	0.2469%	0.1936%	0.1746%	0.1301%	0.2354%
02 44 01	0.3292%	0.2194%	0.1846%	0.2851%	0.2768%	0.3406%	0.2776%	0.2856%	0.2199%	0.3322%	0.2330%
02 44 16	0.2632%	0.1921%	0.0700%	0.1756%	0.2145%	0.3168%	0.1849%	0.1397%	0.2157%	0.4291%	0.1690%
34 08 03	0.2814%	0.1985%	0.2812%	0.1998%	0.2182%	0.3455%	0.2151%	0.3248%	0.3876%	0.1721%	0.2420%
33 14 03	0.2414%	0.1115%	0.1177%	0.1278%	0.2073%	0.2109%	0.1337%	0.2544%	0.3027%	0.1291%	0.4920%
24 35 04	0.2167%	0.1861%	0.1780%	0.2095%	0.1974%	0.1791%	0.4382%	0.4680%	0.1748%	0.5496%	0.1774%
03 51 11	0.2529%	0.2055%	0.2631%	0.1535%	0.2705%	0.3325%	0.1774%	0.2092%	0.1361%	0.2087%	0.2927%
01 52 15	0.0678%	0.2226%	0.2363%	0.2372%	0.1984%	0.2724%	0.1736%	0.1812%	0.1866%	0.3611%	0.1852%
01 37 10	0.1906%	0.1907%	0.2306%	0.1812%	0.2159%	0.3701%	0.3276%	0.3637%	0.2232%	0.3702%	0.0980%
01 51 04	0.3377%	0.2337%	0.2776%	0.2241%	0.2831%	0.2014%	0.2962%	0.2229%	0.3808%	0.1835%	0.2089%
24 44 13	0.1666%	0.3160%	0.2180%	0.1925%	0.2502%	0.1407%	0.3758%	0.3352%	0.2353%	0.2881%	0.2396%
01 35 04	0.0330%	0.2541%	0.2110%	0.1303%	0.2728%	0.1913%	0.3032%	0.2906%	0.2224%	0.1920%	0.1303%
01 44 07	0.1256%	0.3188%	0.2299%	0.1963%	0.2083%	0.3067%	0.1344%	0.2158%	0.2645%	0.0853%	0.1961%
24 35 07	0.1906%	0.2010%	0.2035%	0.0822%	0.2088%	0.2335%	0.2991%	0.0893%	0.3037%	0.2074%	0.2568%
24 44 07	0.1423%	0.2171%	0.3016%	0.1841%	0.2473%	0.2100%	0.1955%	0.2196%	0.2007%	0.3092%	0.2276%
02 15 01	0.2540%	0.2711%	0.0581%	0.1860%	0.1502%	0.3729%	0.3197%	0.3441%	0.2508%	0.3111%	0.1823%
26 38 04	0.1227%	0.1198%	0.0869%	0.1036%	0.2676%	0.2788%	0.1510%	0.0947%	0.2335%	0.1474%	0.2553%
24 15 11	0.2158%	0.1679%	0.2492%	0.3805%	0.2618%	0.2261%	0.3772%	0.3052%	0.2021%	0.2415%	0.1253%
68 51 13	0.2502%	0.2340%	0.4393%	0.2119%	0.2124%	0.1903%	0.3075%	0.1564%	0.1361%	0.2993%	0.0784%
02 40 13	0.2129%	0.2243%	0.2844%	0.3468%	0.1444%	0.1529%	0.1481%	0.2973%	0.3286%	0.1949%	0.1348%

<u>Supplementary material</u>: 3.2.3.1.1 Results/HLA frequency analysis/Haplotype frequencies/In the CEDACE registry, NUTS II Regions and districts/ Three *loci*, low-resolution haplotype frequencies

Haplotype	Viana do Castelo	Braga	Vila Real	Bragança	Porto	Aveiro	Viseu	Guarda	Coimbra	Castelo Branco	Leiria
02 44 12	0.1745%	0.3315%	0.1366%	0.2306%	0.2522%	0.2706%	0.1832%	0.0938%	0.1338%	0.1810%	0.1476%
02 50 04	0.4132%	0.2277%	0.0785%	0.1346%	0.1920%	0.1527%	0.2223%	0.1867%	0.2728%	0.1787%	0.3813%
03 18 13	0.0279%	0.1923%	0.2820%	0.0000%	0.4270%	0.4553%	0.1474%	0.0833%	0.1143%	0.0098%	0.1076%
01 08 07	0.7910%	0.2969%	0.1903%	0.1243%	0.2324%	0.1318%	0.2102%	0.0731%	0.1605%	0.1074%	0.3375%
23 49 13	0.2325%	0.2425%	0.3485%	0.4894%	0.2328%	0.0982%	0.1473%	0.2955%	0.2460%	0.2135%	0.1276%
32 40 13	0.0958%	0.1410%	0.1547%	0.1476%	0.1573%	0.2040%	0.3430%	0.1238%	0.2300%	0.1785%	0.2465%
01 15 13	0.6151%	0.2552%	0.2098%	0.1378%	0.1705%	0.2322%	0.3985%	0.0817%	0.2159%	0.1454%	0.2024%
24 35 13	0.1371%	0.1812%	0.1987%	0.1170%	0.2378%	0.2373%	0.1487%	0.1583%	0.1219%	0.1460%	0.0818%
02 50 13	0.2526%	0.1188%	0.1979%	0.1547%	0.1423%	0.3365%	0.1076%	0.1626%	0.3514%	0.0789%	0.3906%
02 39 16	0.1743%	0.1815%	0.2405%	0.1772%	0.1528%	0.1715%	0.2331%	0.1359%	0.2777%	0.0889%	0.2295%
02 15 11	0.0886%	0.1480%	0.1479%	0.1122%	0.1894%	0.2278%	0.2909%	0.3040%	0.2061%	0.4917%	0.1561%
02 58 07	0.0889%	0.1896%	0.1977%	0.0494%	0.1835%	0.1861%	0.2858%	0.2986%	0.2406%	0.2262%	0.2034%
02 51 01	0.2275%	0.2425%	0.4338%	0.4413%	0.2222%	0.1004%	0.1325%	0.3165%	0.1476%	0.2866%	0.1831%
31 51 13	0.1914%	0.2228%	0.1226%	0.0929%	0.1866%	0.2246%	0.1464%	0.2136%	0.3654%	0.1168%	0.1157%
11 35 14	0.1534%	0.1393%	0.1835%	0.0797%	0.2034%	0.2294%	0.2350%	0.0826%	0.2230%	0.0762%	0.1798%
24 35 14	0.2768%	0.2675%	0.1110%	0.0233%	0.1214%	0.0413%	0.0799%	0.1633%	0.1508%	0.0712%	0.1878%
03 08 03	0.1788%	0.1591%	0.4173%	0.2550%	0.1727%	0.2159%	0.2351%	0.2827%	0.2065%	0.1052%	0.1986%
11 49 04	0.0300%	0.0611%	0.2566%	0.1114%	0.2148%	0.1920%	0.2408%	0.2742%	0.1479%	0.0389%	0.1946%
29 44 13	0.2602%	0.1557%	0.3303%	0.1379%	0.2078%	0.1211%	0.1977%	0.1682%	0.2321%	0.2190%	0.1322%
03 07 13	0.1463%	0.0678%	0.1353%	0.1880%	0.1589%	0.1665%	0.2765%	0.4349%	0.2701%	0.1392%	0.1877%
32 35 04	0.3703%	0.1053%	0.2210%	0.2452%	0.2803%	0.1699%	0.0844%	0.1974%	0.2085%	0.3481%	0.0587%
02 49 11	0.0390%	0.1172%	0.2115%	0.1222%	0.0996%	0.0550%	0.1754%	0.2628%	0.1690%	0.2852%	0.6542%
01 08 13	0.1080%	0.1649%	0.0985%	0.2015%	0.1893%	0.2281%	0.1473%	0.0914%	0.3227%	0.0918%	0.2227%
02 27 01	0.1003%	0.2368%	0.1204%	0.1009%	0.2390%	0.1633%	0.1538%	0.2254%	0.1649%	0.2178%	0.1629%
33 44 01	0.1162%	0.0534%	0.2379%	0.1574%	0.1691%	0.4306%	0.1907%	0.0906%	0.2830%	0.0440%	0.2115%
01 44 04	0.3315%	0.2066%	0.1023%	0.0713%	0.1880%	0.1729%	0.1588%	0.2190%	0.1239%	0.3729%	0.2415%
24 35 01	0.1729%	0.1891%	0.1638%	0.4336%	0.1614%	0.1877%	0.1209%	0.0826%	0.1429%	0.1022%	0.2801%
33 14 13	0.1735%	0.1738%	0.1632%	0.1530%	0.2364%	0.1737%	0.2028%	0.0683%	0.1160%	0.2501%	0.1349%
31 51 04	0.2371%	0.1754%	0.1125%	0.1114%	0.1709%	0.2300%	0.1650%	0.1639%	0.1652%	0.2143%	0.1595%
29 44 04	0.1219%	0.2180%	0.2817%	0.0823%	0.1785%	0.2620%	0.0790%	0.1889%	0.1648%	0.2609%	0.1350%
02 07 01	0.0991%	0.1278%	0.0868%	0.1113%	0.1053%	0.3189%	0.3502%	0.0780%	0.2353%	0.1442%	0.1587%
29 15 13	0.2384%	0.1000%	0.1339%	0.0907%	0.1349%	0.1393%	0.1465%	0.0448%	0.1350%	0.4885%	0.0831%
23 44 01	0.1048%	0.1397%	0.1171%	0.1086%	0.2086%	0.1489%	0.2200%	0.2069%	0.2126%	0.5372%	0.1215%
23 44 13	0.2134%	0.2675%	0.1097%	0.0667%	0.1935%	0.1305%	0.1064%	0.1512%	0.1408%	0.1937%	0.2447%
24 51 11	0.0566%	0.1426%	0.1593%	0.1334%	0.2006%	0.2853%	0.1495%	0.2403%	0.1062%	0.1237%	0.2940%

Supplementary material: 3.2.3.1.1 Results/HLA frequency analysis/Haplotype frequencies/In the CEDACE registry, NUTS II Regions and districts/ Three *loci*, low-resolution haplotype frequencies

Haplotype	Viana do Castelo	Braga	Vila Real	Bragança	Porto	Aveiro	Viseu	Guarda	Coimbra	Castelo Branco	Leiria
03 07 04	0.1504%	0.1972%	0.2336%	0.3263%	0.1665%	0.1772%	0.2659%	0.0452%	0.1949%	0.1382%	0.1556%
68 15 15	0.1412%	0.1909%	0.2116%	0.0487%	0.1177%	0.3203%	0.1793%	0.0796%	0.1664%	0.4288%	0.2768%
02 41 13	0.1511%	0.1980%	0.2390%	0.1918%	0.1612%	0.0663%	0.2884%	0.2916%	0.1755%	0.2637%	0.1045%
02 35 14	0.2110%	0.2074%	0.1196%	0.0000%	0.1938%	0.1134%	0.1039%	0.0991%	0.0881%	0.1425%	0.2273%
02 51 14	0.0842%	0.0920%	0.0966%	0.0835%	0.1302%	0.0595%	0.1047%	0.0261%	0.1179%	0.1012%	0.2701%
24 45 10	0.2033%	0.0923%	0.1605%	0.2173%	0.1822%	0.1124%	0.1861%	0.2155%	0.1308%	0.2176%	0.0990%
01 08 11	0.0331%	0.1207%	0.1712%	0.1792%	0.1384%	0.2132%	0.1612%	0.3437%	0.1459%	0.2606%	0.1483%
02 15 07	0.2069%	0.3855%	0.3198%	0.1604%	0.1752%	0.1255%	0.1151%	0.1792%	0.1456%	0.1438%	0.0551%
11 44 13	0.1665%	0.0726%	0.1229%	0.2646%	0.0922%	0.2669%	0.2158%	0.1923%	0.2179%	0.3964%	0.1632%
02 44 08	0.1704%	0.1854%	0.1211%	0.1370%	0.2173%	0.1233%	0.2602%	0.0768%	0.1774%	0.2201%	0.1058%
24 27 04	0.1410%	0.1376%	0.0823%	0.0441%	0.1553%	0.1796%	0.1331%	0.1140%	0.1954%	0.2895%	0.2656%
33 14 07	0.0722%	0.1871%	0.2215%	0.0953%	0.2517%	0.0625%	0.2273%	0.2601%	0.1219%	0.0313%	0.1393%
02 51 16	0.3954%	0.0968%	0.1329%	0.2077%	0.1487%	0.1262%	0.0666%	0.1680%	0.2002%	0.1270%	0.1105%
24 50 04	0.2664%	0.1410%	0.1581%	0.2755%	0.1410%	0.1170%	0.1799%	0.1075%	0.3032%	0.2114%	0.1533%
11 35 13	0.1138%	0.1346%	0.1372%	0.1071%	0.0888%	0.1747%	0.2840%	0.1946%	0.2461%	0.0526%	0.2232%
03 35 15	0.0508%	0.1453%	0.1901%	0.2631%	0.3284%	0.0567%	0.1383%	0.1160%	0.0881%	0.0950%	0.1003%
03 07 07	0.1227%	0.2461%	0.1258%	0.1940%	0.1833%	0.1305%	0.1208%	0.1346%	0.1433%	0.0696%	0.2169%
02 14 03	0.0908%	0.1077%	0.0963%	0.4276%	0.0849%	0.1381%	0.1355%	0.1753%	0.1337%	0.4797%	0.1882%
02 07 13	0.1207%	0.1489%	0.0537%	0.0289%	0.1036%	0.2942%	0.1058%	0.0521%	0.2623%	0.0608%	0.2246%
03 51 04	0.0381%	0.1149%	0.1621%	0.1713%	0.2094%	0.1706%	0.1944%	0.1185%	0.1407%	0.3107%	0.2449%
02 27 13	0.1544%	0.1620%	0.0787%	0.1293%	0.1449%	0.1764%	0.1116%	0.1217%	0.2034%	0.1106%	0.1867%
11 14 01	0.2277%	0.2778%	0.0709%	0.0788%	0.2010%	0.1149%	0.1109%	0.1062%	0.1310%	0.1183%	0.0743%
02 58 13	0.0577%	0.1931%	0.1768%	0.1986%	0.0970%	0.0685%	0.2711%	0.2149%	0.0935%	0.1060%	0.1724%
11 35 07	0.1267%	0.1414%	0.0475%	0.1026%	0.1237%	0.1068%	0.2031%	0.2535%	0.1263%	0.0622%	0.1930%
03 44 07	0.2630%	0.0954%	0.2155%	0.1134%	0.1355%	0.1395%	0.1571%	0.1657%	0.1902%	0.0699%	0.2256%
32 49 04	0.0901%	0.1420%	0.1897%	0.1680%	0.1534%	0.1167%	0.1564%	0.2665%	0.0933%	0.0744%	0.1347%
11 35 04	0.0657%	0.1204%	0.1815%	0.1424%	0.1946%	0.1902%	0.2276%	0.1630%	0.1059%	0.1303%	0.1084%
24 35 15	0.0230%	0.1023%	0.1111%	0.1965%	0.2044%	0.1334%	0.0726%	0.1643%	0.1614%	0.2785%	0.1118%
02 49 01	0.0406%	0.2479%	0.2932%	0.1753%	0.1581%	0.0756%	0.1706%	0.1549%	0.1212%	0.1587%	0.1185%
02 14 07	0.1631%	0.0926%	0.1969%	0.0219%	0.0826%	0.0764%	0.0753%	0.0492%	0.1986%	0.1826%	0.2912%
11 14 07	0.0663%	0.1093%	0.1131%	0.0691%	0.1658%	0.0870%	0.1556%	0.1124%	0.1429%	0.1966%	0.1535%
29 44 11	0.2107%	0.2120%	0.1208%	0.1882%	0.1628%	0.1642%	0.1327%	0.0892%	0.1953%	0.2281%	0.0319%
23 14 01	0.4473%	0.1410%	0.0243%	0.2001%	0.1519%	0.1075%	0.2046%	0.1014%	0.1793%	0.0936%	0.1419%
26 44 07	0.1920%	0.1511%	0.1477%	0.1034%	0.1690%	0.1173%	0.1789%	0.0848%	0.0973%	0.0673%	0.1070%
01 35 11	0.0272%	0.1797%	0.0208%	0.1026%	0.1083%	0.2235%	0.2667%	0.1650%	0.1824%	0.0718%	0.1200%

Supplementary material: 3.2.3.1.1 Results/HLA frequency analysis/Haplotype frequencies/In the CEDACE registry, NUTS II Regions and districts/ Three *loci*, low-resolution haplotype frequencies

**Haplotype**	**Viana do Castelo**	**Braga**	**Vila Real**	**Bragança**	**Porto**	**Aveiro**	**Viseu**	**Guarda**	**Coimbra**	**Castelo Branco**	**Leiria**
11 27 01	0.1958%	0.1877%	0.2165%	0.2072%	0.1552%	0.1500%	0.1543%	0.0177%	0.1414%	0.0685%	0.1885%
02 38 13	0.3349%	0.1447%	0.1225%	0.1129%	0.1185%	0.1506%	0.1964%	0.1069%	0.1736%	0.1056%	0.0810%
68 51 07	0.1408%	0.1028%	0.3062%	0.0349%	0.1811%	0.1903%	0.1494%	0.0531%	0.1393%	0.0703%	0.1888%
01 35 13	0.0916%	0.1158%	0.1394%	0.2893%	0.1389%	0.1458%	0.2315%	0.2845%	0.0994%	0.0309%	0.1315%
02 50 03	0.0877%	0.1120%	0.2224%	0.1533%	0.1971%	0.1152%	0.1357%	0.2533%	0.2251%	0.0190%	0.0481%
03 35 07	0.0502%	0.2174%	0.0923%	0.0000%	0.2239%	0.0527%	0.1669%	0.0894%	0.0838%	0.0302%	0.0869%
32 14 01	0.0618%	0.1139%	0.2402%	0.0996%	0.1869%	0.1375%	0.1001%	0.2027%	0.0774%	0.2693%	0.0980%
02 35 04	0.3999%	0.1171%	0.0675%	0.0837%	0.0918%	0.0637%	0.1006%	0.2529%	0.1195%	0.2492%	0.1436%
24 51 04	0.0891%	0.1180%	0.1442%	0.0000%	0.1162%	0.3019%	0.1403%	0.2725%	0.0881%	0.1498%	0.0688%
02 51 03	0.1156%	0.1143%	0.1393%	0.0726%	0.1141%	0.1666%	0.1144%	0.1932%	0.1552%	0.0811%	0.1130%

Supplementary Table 7. Frequencies of the 150 most frequent L3G haplotypes according to District, in Districts from the NUTS II Regions of North and Center. Dataset: **L3D**.

<u>Supplementary material</u>: 3.2.3.1.1 Results/HLA frequency analysis/Haplotype frequencies/In the CEDACE registry, NUTS II Regions and districts/ Three *loci*, low-resolution haplotype frequencies

Haplotype	Lisboa	Santarém	Portalegre	Setúbal	Évora	Beja	Faro	Madeira	Açores
01 08 03	2.7758%	3.1302%	2.8517%	2.6914%	2.4245%	2.4868%	2.6829%	2.9221%	3.0868%
29 44 07	1.9617%	1.9769%	1.9556%	1.8996%	1.8530%	1.5484%	1.9212%	2.2219%	2.0739%
02 44 04	1.4644%	1.4618%	1.3695%	1.6354%	1.5718%	1.2585%	1.6644%	1.1215%	1.3326%
33 14 01	1.1197%	1.2594%	1.1746%	1.1135%	1.2344%	1.1043%	1.0978%	2.3735%	0.8051%
03 07 15	1.0984%	1.1846%	0.9992%	1.0384%	1.0178%	1.0846%	1.0223%	1.2351%	1.2557%
02 44 07	1.0067%	0.9271%	1.1026%	1.0683%	1.0906%	0.9743%	1.2716%	1.2355%	0.9351%
03 35 01	1.0048%	1.1832%	1.1569%	0.9705%	1.0522%	1.2814%	1.2197%	0.3580%	0.5198%
02 44 13	0.9267%	0.9700%	0.9151%	0.8828%	0.9537%	1.2396%	1.3052%	1.2168%	1.1409%
23 44 07	0.8585%	0.8430%	0.7858%	0.7463%	0.5737%	0.6222%	0.6619%	0.9768%	0.7483%
02 51 08	0.8714%	0.9351%	0.7415%	0.9239%	0.6231%	0.4577%	0.4611%	0.6134%	0.9169%
11 35 01	0.9215%	0.7623%	0.7718%	1.0023%	0.8384%	0.9311%	1.1744%	1.0605%	1.1270%
02 50 07	0.7276%	0.9882%	0.8260%	0.7680%	0.8326%	0.5487%	0.4340%	1.2929%	0.8045%
02 51 11	0.8775%	0.9340%	1.2321%	0.8879%	0.9275%	0.9835%	0.9193%	0.4939%	0.7406%
02 51 13	0.7130%	0.8229%	1.0609%	0.6818%	0.6625%	1.1038%	0.9382%	0.8239%	0.6351%
02 07 15	0.6817%	0.6187%	0.5035%	0.5588%	0.6855%	0.4126%	0.5807%	0.9755%	1.0655%
24 35 11	0.7500%	0.6662%	0.6981%	0.7595%	0.7183%	0.8071%	0.6841%	0.8432%	0.7916%
02 18 11	0.6671%	0.7317%	0.7934%	0.7097%	1.1646%	1.1284%	0.8028%	1.1196%	0.4217%
01 57 07	0.6071%	0.4907%	0.7530%	0.5239%	0.7281%	0.6168%	0.5473%	0.3636%	0.8513%
02 14 01	0.5344%	0.5293%	0.6422%	0.6435%	0.5825%	1.0598%	0.8410%	0.6229%	0.6625%
02 51 07	0.5838%	0.7764%	0.7289%	0.5103%	0.5531%	0.5274%	0.4541%	0.2710%	0.4552%
30 18 03	0.5817%	0.5727%	0.8239%	0.7090%	0.8623%	0.9884%	1.0510%	0.3022%	0.4536%
02 08 03	0.5089%	0.3548%	0.5374%	0.4681%	0.3746%	0.4857%	0.5601%	0.4323%	0.4619%
02 18 03	0.5618%	0.5933%	0.6100%	0.6643%	0.6825%	0.9036%	0.9040%	0.1396%	0.5508%
02 35 11	0.4375%	0.2970%	0.3882%	0.4864%	0.6274%	0.4923%	0.4299%	0.9643%	0.3770%
02 51 04	0.4944%	0.5620%	0.5989%	0.5700%	0.5723%	0.6809%	0.6146%	0.3374%	0.1885%
02 15 04	0.4630%	0.4778%	0.3249%	0.4643%	0.4126%	0.4228%	0.3953%	0.5552%	0.6157%
24 14 01	0.4206%	0.3187%	0.3870%	0.3350%	0.4171%	0.1433%	0.4609%	0.6364%	0.3002%
02 35 01	0.4204%	0.3265%	0.3630%	0.3990%	0.2496%	1.0028%	0.4353%	0.4679%	0.4480%
24 07 15	0.4098%	0.4813%	0.2104%	0.3439%	0.3000%	0.5206%	0.3574%	0.4989%	0.4384%
26 38 13	0.3941%	0.4962%	0.4413%	0.4172%	0.6836%	0.3209%	0.3462%	0.9088%	0.4942%
24 08 03	0.3858%	0.3594%	0.1440%	0.3313%	0.4540%	0.3279%	0.3842%	0.5168%	0.4281%
03 14 01	0.3831%	0.4555%	0.4329%	0.4389%	0.4853%	0.3405%	0.5461%	0.3797%	0.3249%
02 35 03	0.4123%	0.5556%	0.5516%	0.3927%	0.2681%	0.4407%	0.2466%	0.2512%	0.5140%
02 13 07	0.4699%	0.3389%	0.2452%	0.3012%	0.2983%	0.5932%	0.4662%	0.3985%	0.2358%
02 44 11	0.3724%	0.2388%	0.3353%	0.3393%	0.4410%	0.4335%	0.3107%	0.3598%	0.4178%
30 13 07	0.3550%	0.2585%	0.3220%	0.3764%	0.5267%	0.6047%	0.4526%	0.0831%	0.4076%

<u>Supplementary material</u>: 3.2.3.1.1 Results/HLA frequency analysis/Haplotype frequencies/In the CEDACE registry, NUTS II Regions and districts/ Three *loci*, low-resolution haplotype frequencies

Haplotype	Lisboa	Santarém	Portalegre	Setúbal	Évora	Beja	Faro	Madeira	Açores
03 35 11	0.3751%	0.7149%	0.5480%	0.3494%	0.2363%	0.2989%	0.3337%	0.2516%	0.2686%
24 15 13	0.2925%	0.3907%	0.5885%	0.3171%	0.3790%	0.4425%	0.2774%	0.4859%	0.4614%
25 18 15	0.3524%	0.2573%	0.2340%	0.3192%	0.1609%	0.4032%	0.3475%	0.3082%	0.1636%
68 53 13	0.3502%	0.4811%	0.4032%	0.3456%	0.2738%	0.3452%	0.3084%	0.1818%	0.6308%
02 57 07	0.2831%	0.2110%	0.2146%	0.2665%	0.2156%	0.2034%	0.1916%	0.1126%	0.3376%
02 44 15	0.2652%	0.2683%	0.2939%	0.2444%	0.2161%	0.2352%	0.2191%	0.1595%	0.4382%
03 35 13	0.2565%	0.2293%	0.2829%	0.2892%	0.2621%	0.2323%	0.2208%	0.2505%	0.2337%
32 14 07	0.2595%	0.2767%	0.2467%	0.3175%	0.4217%	0.1342%	0.1571%	0.1028%	0.3051%
31 40 04	0.2664%	0.2578%	0.1486%	0.2893%	0.2359%	0.2118%	0.2773%	0.3788%	0.3507%
02 51 15	0.2755%	0.1836%	0.1966%	0.2988%	0.3089%	0.4266%	0.2474%	0.1330%	0.2141%
02 35 13	0.2716%	0.3036%	0.2972%	0.2482%	0.1208%	0.2559%	0.4220%	0.3200%	0.3713%
02 15 13	0.2568%	0.2217%	0.3286%	0.2522%	0.3322%	0.4643%	0.3055%	0.3943%	0.3395%
24 38 13	0.2513%	0.3436%	0.4749%	0.2542%	0.4931%	0.3124%	0.3131%	0.3855%	0.3674%
11 35 11	0.2521%	0.2874%	0.2420%	0.2979%	0.7281%	0.3716%	0.2849%	0.3849%	0.1170%
02 49 04	0.2307%	0.1995%	0.2667%	0.2278%	0.2119%	0.3076%	0.2294%	0.2261%	0.1997%
02 44 01	0.2760%	0.3059%	0.0537%	0.2138%	0.2023%	0.1407%	0.1582%	0.2525%	0.2716%
02 44 16	0.2758%	0.2546%	0.2991%	0.3494%	0.2181%	0.4037%	0.4561%	0.0842%	0.1284%
34 08 03	0.2191%	0.1566%	0.1199%	0.2088%	0.1382%	0.2691%	0.1874%	0.4185%	0.2239%
33 14 03	0.2324%	0.3696%	0.1396%	0.2213%	0.2389%	0.3431%	0.2912%	0.5397%	0.1860%
24 35 04	0.2500%	0.2587%	0.2686%	0.3089%	0.2302%	0.3112%	0.1932%	0.1251%	0.0866%
03 51 11	0.2087%	0.3250%	0.2026%	0.2045%	0.2113%	0.1584%	0.3085%	0.1441%	0.2148%
01 52 15	0.2652%	0.2711%	0.2325%	0.2808%	0.2455%	0.3653%	0.2476%	0.3035%	0.1424%
01 37 10	0.2279%	0.2468%	0.1819%	0.2408%	0.1987%	0.3820%	0.2089%	0.0866%	0.3065%
01 51 04	0.1757%	0.2282%	0.1048%	0.2189%	0.1546%	0.0721%	0.2063%	0.1309%	0.1305%
24 44 13	0.1963%	0.1950%	0.1122%	0.1975%	0.2118%	0.4813%	0.2748%	0.1895%	0.3181%
01 35 04	0.2013%	0.1737%	0.2393%	0.2468%	0.2957%	0.5147%	0.2031%	0.0907%	0.0889%
01 44 07	0.2408%	0.1335%	0.0521%	0.1912%	0.2829%	0.0168%	0.0754%	0.0695%	0.1726%
24 35 07	0.2078%	0.2247%	0.3668%	0.2019%	0.2035%	0.1184%	0.0650%	0.2772%	0.2221%
24 44 07	0.2190%	0.1344%	0.3031%	0.1883%	0.0713%	0.3404%	0.2334%	0.1371%	0.1675%
02 15 01	0.2021%	0.0929%	0.2409%	0.2192%	0.0535%	0.0348%	0.2148%	0.0681%	0.1821%
26 38 04	0.2057%	0.2645%	0.1643%	0.2270%	0.1510%	0.1744%	0.3153%	0.1667%	0.2276%
24 15 11	0.1729%	0.1597%	0.1327%	0.2384%	0.2580%	0.2031%	0.1604%	0.3204%	0.2658%
68 51 13	0.2387%	0.1873%	0.3419%	0.1936%	0.0773%	0.2867%	0.1179%	0.5030%	0.1664%
02 40 13	0.2551%	0.2323%	0.1763%	0.2652%	0.2732%	0.2864%	0.2124%	0.1482%	0.2995%
02 44 12	0.1932%	0.2730%	0.1367%	0.1586%	0.1736%	0.0636%	0.0953%	0.0736%	0.0962%
02 50 04	0.1745%	0.3200%	0.2427%	0.1404%	0.1973%	0.2509%	0.1864%	0.1807%	0.1572%

<u>Supplementary material</u>: 3.2.3.1.1 Results/HLA frequency analysis/Haplotype frequencies/In the CEDACE registry, NUTS II Regions and districts/ Three *loci*, low-resolution haplotype frequencies

Haplotype	Lisboa	Santarém	Portalegre	Setúbal	Évora	Beja	Faro	Madeira	Açores
03 18 13	0.1096%	0.1592%	0.1967%	0.1380%	0.1654%	0.0734%	0.0780%	0.0623%	0.2491%
01 08 07	0.1743%	0.1484%	0.1209%	0.1313%	0.1645%	0.1099%	0.1217%	0.2473%	0.0860%
23 49 13	0.2069%	0.1979%	0.2267%	0.1882%	0.3468%	0.0789%	0.2182%	0.1487%	0.2100%
32 40 13	0.1832%	0.2626%	0.4100%	0.2303%	0.1297%	0.3358%	0.4559%	0.2862%	0.0608%
01 15 13	0.1929%	0.1790%	0.0000%	0.1651%	0.0954%	0.0763%	0.1649%	0.0839%	0.1227%
24 35 13	0.2128%	0.1961%	0.2647%	0.2928%	0.2539%	0.1946%	0.4516%	0.2853%	0.0037%
02 50 13	0.1773%	0.2253%	0.1831%	0.2036%	0.2645%	0.1371%	0.1551%	0.0782%	0.0541%
02 39 16	0.1902%	0.1758%	0.3729%	0.2424%	0.2180%	0.5152%	0.2148%	0.1433%	0.2358%
02 15 11	0.2100%	0.1936%	0.1377%	0.2177%	0.4190%	0.0837%	0.1029%	0.0795%	0.1649%
02 58 07	0.1887%	0.2817%	0.1174%	0.2184%	0.1867%	0.1316%	0.0829%	0.2282%	0.1157%
02 51 01	0.1848%	0.1954%	0.1185%	0.1632%	0.1132%	0.1318%	0.1784%	0.2535%	0.2884%
31 51 13	0.1641%	0.2641%	0.0980%	0.2185%	0.1160%	0.2320%	0.2666%	0.0154%	0.1652%
11 35 14	0.1940%	0.2143%	0.2120%	0.1731%	0.3894%	0.1246%	0.2048%	0.0819%	0.0236%
24 35 14	0.2813%	0.1323%	0.2548%	0.2490%	0.2479%	0.4133%	0.2088%	0.0084%	0.3086%
03 08 03	0.1851%	0.1417%	0.1640%	0.1820%	0.1889%	0.1768%	0.1181%	0.2943%	0.2672%
11 49 04	0.2090%	0.1357%	0.1493%	0.2236%	0.1527%	0.3445%	0.3694%	0.1297%	0.0619%
29 44 13	0.1711%	0.1604%	0.2408%	0.2279%	0.1073%	0.1555%	0.1469%	0.4642%	0.2717%
03 07 13	0.1936%	0.2022%	0.1621%	0.1864%	0.2473%	0.2657%	0.0994%	0.0635%	0.2583%
32 35 04	0.1615%	0.1782%	0.6318%	0.1612%	0.2091%	0.2232%	0.1335%	0.0409%	0.0561%
02 49 11	0.1966%	0.2970%	0.2320%	0.1281%	0.1938%	0.1470%	0.1503%	0.3473%	0.4691%
01 08 13	0.1701%	0.1556%	0.2466%	0.2017%	0.1263%	0.1355%	0.0796%	0.0712%	0.2033%
02 27 01	0.1565%	0.1331%	0.1753%	0.1829%	0.2528%	0.2641%	0.1098%	0.1649%	0.1247%
33 44 01	0.1669%	0.1055%	0.0422%	0.1616%	0.1378%	0.2332%	0.1972%	0.0473%	0.0958%
01 44 04	0.1563%	0.2588%	0.2623%	0.1388%	0.1553%	0.3414%	0.0665%	0.0042%	0.2787%
24 35 01	0.1550%	0.2331%	0.1701%	0.2089%	0.1963%	0.1806%	0.3343%	0.1475%	0.2742%
33 14 13	0.1602%	0.1949%	0.2500%	0.1865%	0.2784%	0.1015%	0.1166%	0.2094%	0.1919%
31 51 04	0.1849%	0.1309%	0.3616%	0.1505%	0.2740%	0.0926%	0.1117%	0.1086%	0.0524%
29 44 04	0.1562%	0.1677%	0.1730%	0.2130%	0.1929%	0.0695%	0.1381%	0.0770%	0.0000%
02 07 01	0.1680%	0.1163%	0.2132%	0.1675%	0.2040%	0.0876%	0.1832%	0.4405%	0.4397%
29 15 13	0.2114%	0.4247%	0.2608%	0.1710%	0.2700%	0.0834%	0.1160%	0.4859%	0.1472%
23 44 01	0.1817%	0.1347%	0.2329%	0.1347%	0.1229%	0.0000%	0.0621%	0.3281%	0.1369%
23 44 13	0.1566%	0.2245%	0.2436%	0.1423%	0.1086%	0.1607%	0.1013%	0.0646%	0.0958%
24 51 11	0.1705%	0.1266%	0.0883%	0.1672%	0.1595%	0.2685%	0.0959%	0.1030%	0.0000%
03 07 04	0.1759%	0.0988%	0.1530%	0.1729%	0.1838%	0.1792%	0.1023%	0.1179%	0.1339%
68 15 15	0.1652%	0.1800%	0.1127%	0.1409%	0.0813%	0.0503%	0.0746%	0.0673%	0.0387%
02 41 13	0.1511%	0.2481%	0.2851%	0.2130%	0.1431%	0.2752%	0.3082%	0.0806%	0.1205%

<u>Supplementary material</u>: 3.2.3.1.1 Results/HLA frequency analysis/Haplotype frequencies/In the CEDACE registry, NUTS II Regions and districts/ Three *loci*, low-resolution haplotype frequencies

Haplotype	Lisboa	Santarém	Portalegre	Setúbal	Évora	Beja	Faro	Madeira	Açores
02 35 14	0.1799%	0.3228%	0.1306%	0.1671%	0.2195%	0.0962%	0.0488%	0.2163%	0.1665%
02 51 14	0.2377%	0.2830%	0.1379%	0.1873%	0.1517%	0.3195%	0.1885%	0.1779%	0.1664%
24 45 10	0.1854%	0.2191%	0.2124%	0.2028%	0.1397%	0.1140%	0.1800%	0.0587%	0.2795%
01 08 11	0.1609%	0.2665%	0.1262%	0.1636%	0.2311%	0.2926%	0.2037%	0.3843%	0.1504%
02 15 07	0.1474%	0.1713%	0.2093%	0.1106%	0.2509%	0.0891%	0.1258%	0.1048%	0.0527%
11 44 13	0.1611%	0.1238%	0.2422%	0.2077%	0.1003%	0.0726%	0.1483%	0.3172%	0.2783%
02 44 08	0.1449%	0.1468%	0.0424%	0.1606%	0.0008%	0.2150%	0.1357%	0.0762%	0.1401%
24 27 04	0.1625%	0.2282%	0.1261%	0.1467%	0.1331%	0.1599%	0.1185%	0.0627%	0.1361%
33 14 07	0.1377%	0.1234%	0.0932%	0.1298%	0.1966%	0.1455%	0.0873%	0.5107%	0.2260%
02 51 16	0.1774%	0.1307%	0.2034%	0.1730%	0.0706%	0.2977%	0.3828%	0.0451%	0.3553%
24 50 04	0.1734%	0.1675%	0.1354%	0.1766%	0.0431%	0.0829%	0.1276%	0.0000%	0.0738%
11 35 13	0.1693%	0.2327%	0.0784%	0.2152%	0.0661%	0.3186%	0.2355%	0.0163%	0.0000%
03 35 15	0.1186%	0.1202%	0.1975%	0.1638%	0.1832%	0.0935%	0.2430%	0.2190%	0.0259%
03 07 07	0.1266%	0.1850%	0.0965%	0.2005%	0.0872%	0.2327%	0.1613%	0.1982%	0.0419%
02 14 03	0.1915%	0.2076%	0.1701%	0.2228%	0.2586%	0.2178%	0.1041%	0.1597%	0.1280%
02 07 13	0.1508%	0.2098%	0.0689%	0.1328%	0.2113%	0.3437%	0.1408%	0.1400%	0.2109%
03 51 04	0.1430%	0.1414%	0.2521%	0.1445%	0.1035%	0.0000%	0.0950%	0.0304%	0.0651%
02 27 13	0.1621%	0.1490%	0.1110%	0.1602%	0.1130%	0.3897%	0.2282%	0.0369%	0.0883%
11 14 01	0.1166%	0.1125%	0.2089%	0.1931%	0.1442%	0.1303%	0.1332%	0.0978%	0.2804%
02 58 13	0.1654%	0.1858%	0.2123%	0.2060%	0.3609%	0.2423%	0.2639%	0.0965%	0.2447%
11 35 07	0.1777%	0.1863%	0.1551%	0.1274%	0.1695%	0.2284%	0.1037%	0.0725%	0.3350%
03 44 07	0.1590%	0.1354%	0.2016%	0.1408%	0.1700%	0.1295%	0.0762%	0.1469%	0.0816%
32 49 04	0.1516%	0.1490%	0.2387%	0.1793%	0.1608%	0.2630%	0.1291%	0.2917%	0.0751%
11 35 04	0.1693%	0.0794%	0.0448%	0.1657%	0.0835%	0.1234%	0.1429%	0.0542%	0.1387%
24 35 15	0.1621%	0.0983%	0.1485%	0.1754%	0.2111%	0.1703%	0.0673%	0.0905%	0.1592%
02 49 01	0.1418%	0.3106%	0.1522%	0.1042%	0.1860%	0.1102%	0.0801%	0.0325%	0.1299%
02 14 07	0.1864%	0.1546%	0.0277%	0.1971%	0.1390%	0.1951%	0.2879%	0.1495%	0.2251%
11 14 07	0.1468%	0.1817%	0.0910%	0.1588%	0.1140%	0.1458%	0.3292%	0.2131%	0.1538%
29 44 11	0.1144%	0.1192%	0.1495%	0.1853%	0.1936%	0.1215%	0.0990%	0.0210%	0.2797%
23 14 01	0.1573%	0.0847%	0.0161%	0.1107%	0.0404%	0.0585%	0.0721%	0.0242%	0.2124%
26 44 07	0.1582%	0.1038%	0.2523%	0.1251%	0.0711%	0.0991%	0.1210%	0.2460%	0.1596%
01 35 11	0.1311%	0.1278%	0.1332%	0.1457%	0.1730%	0.0988%	0.2525%	0.0928%	0.2854%
11 27 01	0.1332%	0.1293%	0.0230%	0.1025%	0.1676%	0.1465%	0.0665%	0.1923%	0.0619%
02 38 13	0.1270%	0.1090%	0.0531%	0.1961%	0.1412%	0.3562%	0.1087%	0.0602%	0.1617%
68 51 07	0.1260%	0.1432%	0.1846%	0.0994%	0.0703%	0.1749%	0.0895%	0.0150%	0.0692%
01 35 13	0.1480%	0.1335%	0.1953%	0.1563%	0.1745%	0.1219%	0.1458%	0.0324%	0.1768%

Supplementary material: 3.2.3.1.1 Results/HLA frequency analysis/Haplotype frequencies/In the CEDACE registry, NUTS II Regions and districts/ Three *loci*, low-resolution haplotype frequencies

Haplotype	Lisboa	Santarém	Portalegre	Setúbal	Évora	Beja	Faro	Madeira	Açores
02 50 03	0.1310%	0.0697%	0.1135%	0.1380%	0.0274%	0.1887%	0.0943%	0.1528%	0.2381%
03 35 07	0.1069%	0.2424%	0.0748%	0.1148%	0.1433%	0.0604%	0.1235%	0.5161%	0.1270%
32 14 01	0.1391%	0.0429%	0.1785%	0.1260%	0.2247%	0.1777%	0.0988%	0.0677%	0.2031%
02 35 04	0.1847%	0.1401%	0.3630%	0.1695%	0.1506%	0.1300%	0.1721%	0.1004%	0.1882%
24 51 04	0.1551%	0.0966%	0.1552%	0.1516%	0.1747%	0.1901%	0.1820%	0.0699%	0.0997%
02 51 03	0.1384%	0.1259%	0.3350%	0.1771%	0.1278%	0.3213%	0.2871%	0.0171%	0.0948%

Supplementary Table 8. Frequencies of the 150 most frequent L3G haplotypes according to District, in Districts from the NUTS II Regions of the Metropolitan Area of Lisbon, Alentejo, Algarve and the Autonomous Regions of Madeira and Azores. Dataset: **L3D**.

Supplementary material: 3.2.3.1.1 Results/HLA frequency analysis/Haplotype frequencies/In the CEDACE registry, NUTS II Regions and districts/ Three *loci*, low-resolution haplotype frequencies

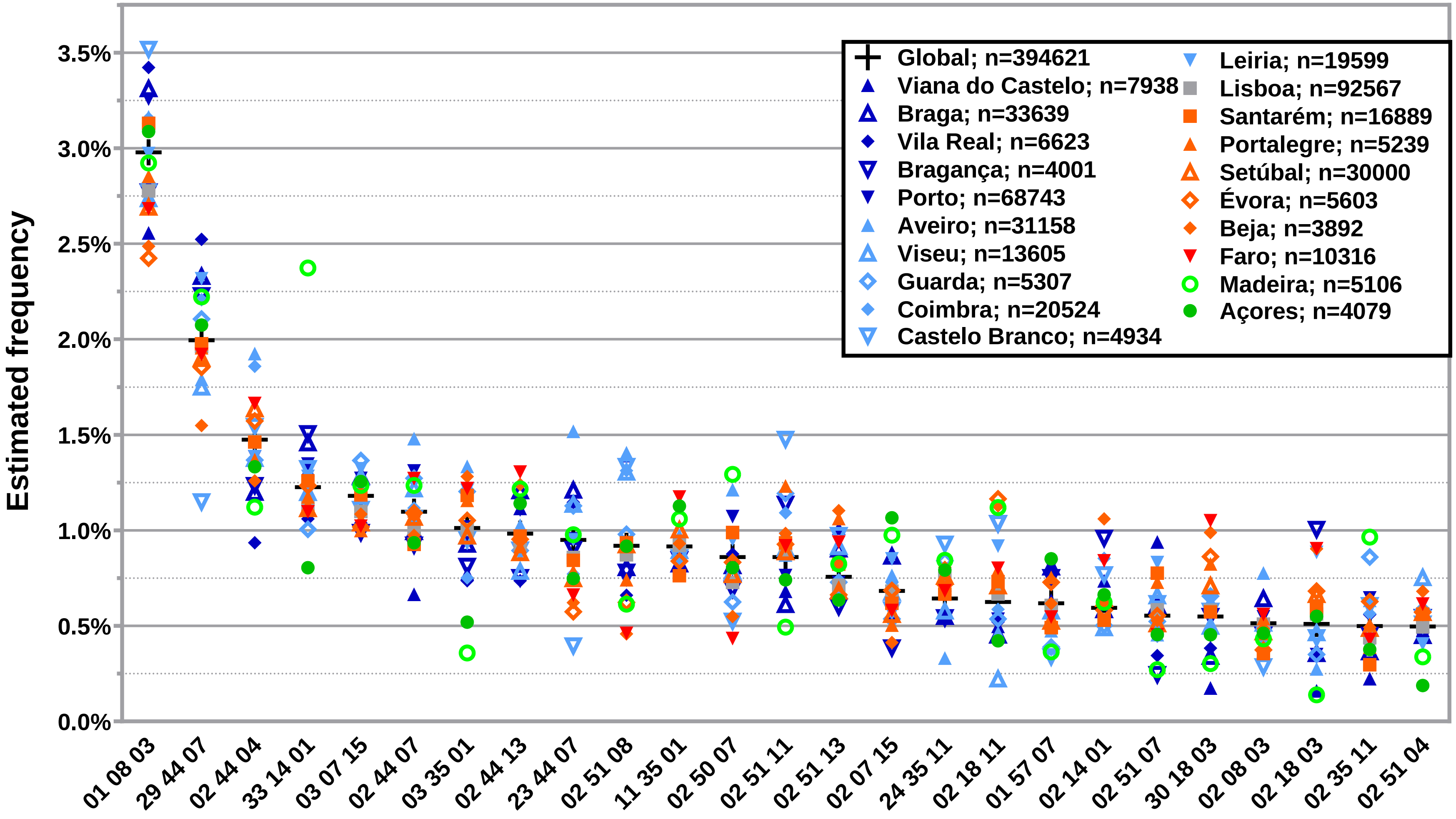

Supplementary Figure 7. Frequency distribution of the 25 most common L3G haplotypes and corresponding frequencies according to District. Dataset: **L3D**.

<u>Supplementary material</u>: 3.2.3.1.3 Results/HLA frequency analysis/Haplotype frequencies/In the CEDACE registry, NUTS II Regions and districts/High-resolution haplotype frequency estimation

Haplotype	Global	North	Center	MA of Lisbon	Alentejo	Algarve	AR of Madeira	AR of Azores
01 08 07 03	2.8810%	3.0614%	2.9017%	2.6872%	2.6207%	2.6967%	2.4005%	3.0918%
29 44 16 07	1.8886%	2.0020%	1.8604%	1.7477%	1.7804%	1.8161%	2.4395%	2.0912%
33 14 08 01	1.1936%	1.3386%	1.0651%	1.0772%	1.0729%	1.0860%	1.8710%	0.6811%
03 07 07 15	1.1558%	1.2493%	1.2210%	1.0102%	0.9945%	1.0439%	1.3704%	1.2401%
02 44 05 04	1.0044%	1.0243%	1.0425%	0.9998%	0.8005%	1.2396%	1.4151%	1.1241%
03 35 04 01	0.9996%	1.0035%	1.0794%	0.9999%	1.0127%	1.1005%	0.1015%	0.3269%
11 35 04 01	0.9053%	0.8816%	0.8782%	0.9484%	0.8886%	0.9918%	1.1279%	1.3115%
23 44 04 07	0.8260%	0.9636%	0.7340%	0.7551%	0.5775%	0.6156%	0.8645%	0.5924%
02 44 05 13	0.8127%	0.9068%	0.6383%	0.7172%	0.9301%	1.0722%	0.8936%	1.4693%
02 50 06 07	0.7725%	0.8312%	0.7950%	0.6942%	0.7497%	0.4322%	1.3058%	0.9006%
02 51 14 08	0.6967%	0.6683%	0.8577%	0.7171%	0.5359%	0.3648%	0.4623%	0.7643%
02 07 07 15	0.6604%	0.6785%	0.6305%	0.6923%	0.6256%	0.6663%	0.8278%	0.8363%
02 14 08 01	0.5265%	0.4910%	0.5600%	0.5300%	0.6787%	0.7507%	0.4799%	0.8597%
30 18 05 03	0.5215%	0.4269%	0.4799%	0.6375%	0.7231%	0.9208%	0.2626%	0.4591%
24 35 04 11	0.5179%	0.4303%	0.5114%	0.6204%	0.7982%	0.6735%	0.8421%	0.6503%
02 08 07 03	0.5154%	0.5552%	0.5105%	0.4759%	0.4074%	0.6605%	0.5473%	0.6464%
02 35 04 11	0.4985%	0.5423%	0.4455%	0.4549%	0.5561%	0.4893%	1.0892%	0.1863%
02 18 07 11	0.4909%	0.3644%	0.5903%	0.5601%	0.8748%	0.6148%	0.9896%	0.3869%
02 44 04 07	0.4751%	0.5753%	0.3853%	0.3918%	0.4067%	0.5869%	0.9204%	0.1110%
02 18 05 03	0.4653%	0.3203%	0.5325%	0.6083%	0.6835%	0.7460%	0.2421%	0.6023%
02 15 03 04	0.4252%	0.4491%	0.4461%	0.4219%	0.3445%	0.3527%	0.3542%	0.4320%
02 35 04 01	0.4156%	0.4357%	0.4066%	0.3889%	0.4670%	0.4502%	0.7168%	0.0125%
24 07 07 15	0.4034%	0.4408%	0.3920%	0.3806%	0.2808%	0.3473%	0.9299%	0.5548%
02 35 04 03	0.3750%	0.3221%	0.5213%	0.4223%	0.4043%	0.2217%	0.0000%	0.3054%
24 08 07 03	0.3743%	0.4285%	0.3198%	0.3440%	0.3187%	0.3019%	0.4237%	0.4253%
26 38 12 13	0.3732%	0.3632%	0.3571%	0.3934%	0.4675%	0.3168%	0.7575%	0.0896%
30 13 06 07	0.3569%	0.4112%	0.2260%	0.3213%	0.4583%	0.4841%	0.0000%	0.3554%
02 13 06 07	0.3522%	0.3376%	0.3599%	0.3744%	0.4288%	0.3728%	0.3653%	0.4235%
25 18 12 15	0.3373%	0.3796%	0.3273%	0.3060%	0.2073%	0.3547%	0.2101%	0.1502%
01 57 07 07	0.3365%	0.3839%	0.2581%	0.3224%	0.3560%	0.2517%	0.1050%	0.5270%
24 14 02 01	0.3316%	0.3583%	0.3592%	0.2727%	0.2812%	0.3915%	0.5252%	0.2937%
02 44 16 07	0.3226%	0.3142%	0.4083%	0.3286%	0.2463%	0.2903%	0.1590%	0.4053%
03 35 04 11	0.3202%	0.2677%	0.3995%	0.3577%	0.3811%	0.4273%	0.3260%	0.2369%
68 53 04 13	0.3027%	0.2436%	0.3487%	0.3580%	0.3812%	0.2924%	0.5017%	0.6108%
01 57 06 07	0.2938%	0.3516%	0.2394%	0.2425%	0.2506%	0.3446%	0.1555%	0.1454%
32 14 08 07	0.2807%	0.3179%	0.2433%	0.2603%	0.2765%	0.1782%	0.1768%	0.2220%
02 49 07 04	0.2736%	0.3351%	0.2038%	0.2253%	0.2734%	0.2080%	0.4398%	0.1788%
03 35 04 13	0.2720%	0.3217%	0.2184%	0.2454%	0.3019%	0.2489%	0.2333%	0.0855%
02 44 16 04	0.2683%	0.2074%	0.3391%	0.2934%	0.3368%	0.4564%	0.3662%	0.2656%
24 38 12 13	0.2641%	0.2160%	0.3071%	0.2634%	0.4471%	0.3982%	0.5511%	0.4274%
31 40 03 04	0.2610%	0.2263%	0.3500%	0.2445%	0.3261%	0.2022%	0.4114%	0.5420%
02 51 15 11	0.2596%	0.2120%	0.3825%	0.2879%	0.2595%	0.2102%	0.2749%	0.3160%
03 14 08 01	0.2565%	0.2712%	0.2187%	0.2385%	0.2928%	0.4771%	0.2928%	0.2581%
02 44 05 11	0.2541%	0.2887%	0.1862%	0.2528%	0.1831%	0.2379%	0.2615%	0.2214%
11 35 04 11	0.2538%	0.2125%	0.2388%	0.2924%	0.5045%	0.2151%	0.3856%	0.1256%
02 35 04 13	0.2457%	0.2429%	0.2609%	0.2531%	0.2459%	0.4859%	0.0000%	0.5786%
24 15 03 13	0.2412%	0.2730%	0.2195%	0.1779%	0.2846%	0.3167%	0.4777%	0.1326%
01 35 04 04	0.2310%	0.2535%	0.2221%	0.2192%	0.2459%	0.1993%	0.0570%	0.1046%
01 37 06 10	0.2297%	0.2245%	0.2537%	0.2262%	0.2617%	0.1792%	0.0526%	0.1877%
02 44 05 07	0.2272%	0.2345%	0.2776%	0.2044%	0.2126%	0.1876%	0.2725%	0.1248%

Supplementary Table 9. Four *loci*, low-resolution frequencies of the 50 most frequent HLA-A/-B/-C/-DRB1 haplotypes in CEDACE and corresponding frequencies according to NUTS II Region. Dataset: **L4R**.

Supplementary material: **3.2.3.1.4 Results/HLA frequency analysis/Haplotype frequencies/In the CEDACE registry, NUTS II Regions and districts/Neighbor-joining trees**

	CEDACE	North	Center	MA of Lisbon	Alentejo	Algarve	AR of Madeira	AR of Azores
**CEDACE**	0.00000	0.00008	0.00004	0.00004	0.00024	0.00039	0.00085	0.00027
**North**	0.00008	0.00000	0.00016	0.00022	0.00059	0.00078	0.00086	0.00035
**Center**	0.00004	0.00016	0.00000	0.00009	0.00028	0.00043	0.00108	0.00036
**MA of Lisbon**	0.00004	0.00022	0.00009	0.00000	0.00011	0.00025	0.00088	0.00029
**Alentejo**	0.00024	0.00059	0.00028	0.00011	0.00000	0.00011	0.00113	0.00054
**Algarve**	0.00039	0.00078	0.00043	0.00025	0.00011	0.00000	0.00129	0.00070
**AR of Madeira**	0.00085	0.00086	0.00108	0.00088	0.00113	0.00129	0.00000	0.00085
**AR of Azores**	0.00027	0.00035	0.00036	0.00029	0.00054	0.00070	0.00085	0.00000

Supplementary Figure 8. Matrix of F_{ST} between Regions, as calculated by AMOVA; heatmap code: blue to red reflecting smaller to greater distances. Dataset: **L3R**.

<u>Supplementary material</u>: 3.2.3.1.4 Results/HLA frequency analysis/Haplotype frequencies/In the CEDACE registry, NUTS II Regions and districts/Neighbor-joining trees

	CEDACE	Viana do Castelo	Braga	Vila Real	Bragança	Porto	Aveiro	Viseu	Guarda	Coimbra	Castelo Branco	Leiria	Lisboa	Santarém	Portalegre	Setúbal	Évora	Beja	Faro	Madeira	Açores
**CEDACE**	0.00000	0.00037	0.00023	0.00027	0.00014	0.00008	0.00021	0.00023	0.00019	0.00018	0.00069	0.00010	0.00002	0.00010	0.00034	0.00010	0.00042	0.00061	0.00039	0.00084	0.00027
**Viana do Castelo**	0.00037	0.00000	0.00016	0.00040	0.00078	0.00035	0.00052	0.00039	0.00080	0.00061	0.00141	0.00061	0.00045	0.00060	0.00109	0.00072	0.00122	0.00151	0.00120	0.00118	0.00061
**Braga**	0.00023	0.00016	0.00000	0.00028	0.00055	0.00019	0.00034	0.00038	0.00071	0.00048	0.00135	0.00037	0.00033	0.00049	0.00095	0.00059	0.00107	0.00135	0.00100	0.00083	0.00042
**Vila Real**	0.00027	0.00040	0.00028	0.00000	0.00050	0.00020	0.00043	0.00048	0.00052	0.00055	0.00140	0.00039	0.00035	0.00050	0.00078	0.00055	0.00091	0.00135	0.00097	0.00107	0.00046
**Bragança**	0.00014	0.00078	0.00055	0.00050	0.00000	0.00033	0.00044	0.00050	0.00017	0.00036	0.00063	0.00019	0.00012	0.00022	0.00035	0.00013	0.00021	0.00044	0.00027	0.00080	0.00034
**Porto**	0.00008	0.00035	0.00019	0.00020	0.00033	0.00000	0.00020	0.00023	0.00037	0.00039	0.00116	0.00023	0.00015	0.00032	0.00063	0.00031	0.00068	0.00106	0.00074	0.00088	0.00036
**Aveiro**	0.00021	0.00052	0.00034	0.00043	0.00044	0.00020	0.00000	0.00022	0.00045	0.00033	0.00100	0.00035	0.00034	0.00041	0.00076	0.00046	0.00102	0.00121	0.00087	0.00132	0.00066
**Viseu**	0.00023	0.00039	0.00038	0.00048	0.00050	0.00023	0.00022	0.00000	0.00039	0.00045	0.00097	0.00052	0.00032	0.00042	0.00072	0.00043	0.00101	0.00114	0.00091	0.00142	0.00053
**Guarda**	0.00019	0.00080	0.00071	0.00052	0.00017	0.00037	0.00045	0.00039	0.00000	0.00042	0.00056	0.00036	0.00017	0.00020	0.00027	0.00015	0.00031	0.00040	0.00035	0.00130	0.00049
**Coimbra**	0.00018	0.00061	0.00048	0.00055	0.00036	0.00039	0.00033	0.00045	0.00042	0.00000	0.00061	0.00021	0.00024	0.00022	0.00053	0.00026	0.00068	0.00065	0.00049	0.00131	0.00050
**Castelo Branco**	0.00069	0.00141	0.00135	0.00140	0.00063	0.00116	0.00100	0.00097	0.00056	0.00061	0.00000	0.00077	0.00063	0.00044	0.00055	0.00052	0.00072	0.00059	0.00068	0.00193	0.00104
**Leiria**	0.00010	0.00061	0.00037	0.00039	0.00019	0.00023	0.00035	0.00052	0.00036	0.00021	0.00077	0.00000	0.00014	0.00012	0.00035	0.00020	0.00040	0.00063	0.00041	0.00083	0.00039
**Lisboa**	0.00002	0.00045	0.00033	0.00035	0.00012	0.00015	0.00034	0.00032	0.00017	0.00024	0.00063	0.00014	0.00000	0.00009	0.00027	0.00005	0.00030	0.00048	0.00031	0.00082	0.00025
**Santarém**	0.00010	0.00060	0.00049	0.00050	0.00022	0.00032	0.00041	0.00042	0.00020	0.00022	0.00044	0.00012	0.00009	0.00000	0.00014	0.00009	0.00031	0.00044	0.00028	0.00110	0.00041
**Portalegre**	0.00034	0.00109	0.00095	0.00078	0.00035	0.00063	0.00076	0.00072	0.00027	0.00053	0.00055	0.00035	0.00027	0.00014	0.00000	0.00017	0.00028	0.00035	0.00023	0.00141	0.00075
**Setúbal**	0.00010	0.00072	0.00059	0.00055	0.00013	0.00031	0.00046	0.00043	0.00015	0.00026	0.00052	0.00020	0.00005	0.00009	0.00017	0.00000	0.00018	0.00030	0.00015	0.00104	0.00041
**Évora**	0.00042	0.00122	0.00107	0.00091	0.00021	0.00068	0.00102	0.00101	0.00031	0.00068	0.00072	0.00040	0.00030	0.00031	0.00028	0.00018	0.00000	0.00033	0.00027	0.00112	0.00071
**Beja**	0.00061	0.00151	0.00135	0.00135	0.00044	0.00106	0.00121	0.00114	0.00040	0.00065	0.00059	0.00063	0.00048	0.00044	0.00035	0.00030	0.00033	0.00000	0.00011	0.00158	0.00097
**Faro**	0.00039	0.00120	0.00100	0.00097	0.00027	0.00074	0.00087	0.00091	0.00035	0.00049	0.00068	0.00041	0.00031	0.00028	0.00023	0.00015	0.00027	0.00011	0.00000	0.00129	0.00070
**Madeira**	0.00084	0.00118	0.00083	0.00107	0.00080	0.00088	0.00132	0.00142	0.00130	0.00131	0.00193	0.00083	0.00082	0.00110	0.00141	0.00104	0.00112	0.00158	0.00129	0.00000	0.00085
**Açores**	0.00027	0.00061	0.00042	0.00046	0.00034	0.00036	0.00066	0.00053	0.00049	0.00050	0.00104	0.00039	0.00025	0.00041	0.00075	0.00041	0.00071	0.00097	0.00070	0.00085	0.00000

Supplementary Figure 9. Matrix of F_{ST} between Districts, as calculated by AMOVA; heatmap code: blue to red reflecting smaller to greater distances. Dataset: **L3D**.

<u>Supplementary material</u>: 3.2.3.2 Results/HLA frequency analysis/Haplotype frequencies/ In African and foreign donors

**Haplotype**	**African**	**Global**
01 08 03	1.3564%	2.9784%
23 49 13	1.0707%	0.2041%
29 44 07	1.0465%	1.9949%
30 42 03	0.9937%	0.0547%
33 15 10	0.9348%	0.0112%
02 35 11	0.8326%	0.4992%
69 15 13	0.8043%	0.0647%
30 08 04	0.8008%	0.0118%
26 08 13	0.7202%	0.0135%
23 15 11	0.6767%	0.0306%
33 14 01	0.6652%	1.2262%
66 53 13	0.6531%	0.0080%
02 51 11	0.6317%	0.8598%
68 53 10	0.5905%	0.0101%
03 58 03	0.5827%	0.0183%
02 35 01	0.5475%	0.4226%
01 37 07	0.5314%	0.0682%
02 35 13	0.5063%	0.2665%
11 35 01	0.4956%	0.9164%
30 35 10	0.4934%	0.0162%
02 07 15	0.4913%	0.6833%
02 50 07	0.4888%	0.8603%
02 44 13	0.4883%	0.9824%
23 44 07	0.4879%	0.9495%
02 53 13	0.4814%	0.0833%
03 35 11	0.4758%	0.3532%
68 58 13	0.4734%	0.0979%
01 08 15	0.4710%	0.1225%
68 53 13	0.4643%	0.3129%
03 07 15	0.4636%	1.1802%
74 15 13	0.4523%	0.0094%
30 53 09	0.4357%	0.0079%
01 49 13	0.4165%	0.0911%
30 14 15	0.4100%	0.0152%
02 15 01	0.4041%	0.2140%
68 35 04	0.4026%	0.0546%
01 08 13	0.3990%	0.1813%
02 44 07	0.3921%	1.0981%
02 18 03	0.3874%	0.5104%
30 18 03	0.3856%	0.5488%
66 58 13	0.3832%	0.0082%
02 44 04	0.3777%	1.4742%
02 45 13	0.3773%	0.0823%
68 15 13	0.3728%	0.0468%
02 14 01	0.3633%	0.5944%
02 51 04	0.3584%	0.4970%
32 15 10	0.3452%	0.0031%
01 52 15	0.3427%	0.2332%
30 35 11	0.3417%	0.0339%
24 35 11	0.3381%	0.6431%

Supplementary Table 10. 3 *loci*, low-resolution frequencies of the 50 most frequent HLA-A/-B/-DRB1 haplotypes in donors of self-declared African ancestry and corresponding frequencies in the CEDACE Registry. Dataset: **L3NW**.

<u>Supplementary material</u>: 3.2.3.2 Results/HLA frequency analysis/Haplotype frequencies/ In African and foreign donors

Haplotype	Cape Verde	Global
23 49 13	1.8278%	0.2041%
69 15 13	1.6331%	0.0647%
23 15 11	1.5511%	0.0306%
02 35 11	1.5017%	0.4992%
33 15 10	1.3820%	0.0112%
26 08 13	1.1285%	0.0135%
30 35 10	1.0812%	0.0162%
03 58 03	0.9983%	0.0183%
30 08 04	0.9983%	0.0118%
66 53 13	0.9659%	0.0080%
68 53 10	0.9103%	0.0101%
01 37 07	0.9075%	0.0682%
30 53 09	0.8978%	0.0079%
01 08 03	0.8957%	2.9784%
30 42 03	0.8319%	0.0547%
02 15 01	0.8294%	0.2140%
02 51 04	0.8247%	0.4970%
02 44 07	0.7416%	1.0981%
02 40 13	0.6656%	0.2113%
02 35 03	0.6656%	0.3962%
31 35 07	0.6656%	0.0205%
03 07 15	0.6494%	1.1802%
01 53 13	0.6429%	0.0171%
03 35 11	0.6417%	0.3532%
30 18 03	0.6225%	0.5488%
01 49 13	0.6142%	0.0911%
30 08 13	0.6097%	0.0052%
11 35 11	0.6020%	0.2614%
02 15 10	0.5844%	0.0193%
02 27 01	0.5824%	0.1796%
23 14 08	0.5824%	0.0052%
23 14 01	0.5816%	0.1436%
03 57 07	0.5693%	0.0614%
32 15 10	0.5686%	0.0031%
23 58 13	0.5652%	0.0557%
68 53 13	0.5572%	0.3129%
33 08 13	0.5551%	0.0085%
23 51 13	0.5513%	0.0102%
02 35 13	0.5324%	0.2665%
30 15 11	0.5145%	0.0166%
02 50 07	0.4992%	0.8603%
01 13 07	0.4992%	0.0654%
30 57 07	0.4992%	0.0103%
68 14 03	0.4992%	0.0063%
02 53 11	0.4936%	0.0085%
03 35 04	0.4926%	0.1319%
03 35 13	0.4811%	0.2691%
68 35 04	0.4777%	0.0546%
33 58 09	0.4733%	0.0013%
11 35 01	0.4575%	0.9164%

Supplementary Table 11. 3 *loci*, low-resolution frequencies of the 50 most frequent HLA-A/-B/-DRB1 haplotypes in donors from Cape Verde and corresponding frequencies in the CEDACE Registry. Dataset: **L3F**.

<u>Supplementary material</u>: 3.2.3.2 Results/HLA frequency analysis/Haplotype frequencies/ In African and foreign donors

**Haplotype**	**Mozambique**	**Global**
02 44 11	2.6119%	0.3624%
01 08 03	2.2388%	2.9784%
33 14 01	1.8657%	1.2262%
02 35 04	1.8657%	0.1405%
02 44 01	1.8150%	0.2553%
02 40 13	1.4925%	0.2113%
02 07 15	1.4925%	0.6833%
01 44 04	1.4925%	0.1770%
33 44 07	1.4266%	0.0739%
02 51 04	1.1194%	0.4970%
24 35 11	1.1194%	0.6431%
23 44 04	1.1194%	0.1314%
02 14 01	1.1194%	0.5944%
24 35 07	1.1194%	0.2162%
24 15 04	1.1194%	0.1084%
68 35 13	1.1194%	0.0672%
29 44 11	1.1194%	0.1446%
02 39 13	1.1194%	0.1338%
11 40 15	1.1194%	0.0085%
02 35 03	0.7463%	0.3962%
02 27 01	0.7463%	0.1796%
02 58 03	0.7463%	0.0434%
03 15 13	0.7463%	0.1367%
30 15 07	0.7463%	0.0063%
30 18 15	0.7463%	0.0460%
24 15 13	0.7463%	0.3493%
68 08 03	0.7463%	0.1208%
30 51 03	0.7463%	0.0041%
01 07 15	0.7463%	0.1006%
24 07 07	0.7463%	0.0381%
24 07 13	0.7463%	0.0727%
02 15 03	0.7463%	0.0721%
29 42 03	0.7463%	0.0009%
02 14 13	0.7463%	0.0486%
03 51 15	0.7463%	0.0845%
32 44 04	0.7463%	0.0837%
26 07 15	0.7463%	0.0480%
32 44 12	0.7463%	0.0190%
33 08 03	0.7463%	0.0163%
29 35 07	0.7463%	0.0119%
23 57 13	0.7463%	0.0078%
29 18 11	0.7463%	0.0053%
26 15 07	0.7463%	0.0033%
11 58 01	0.7463%	0.0000%
68 08 07	0.7462%	0.0013%
03 35 04	0.6297%	0.1319%
03 44 07	0.4391%	0.1495%
33 35 04	0.4391%	0.0071%
02 44 04	0.4238%	1.4742%
03 35 01	0.4238%	1.0120%

Supplementary Table 12. 3 *loci*, low-resolution frequencies of the 50 most frequent HLA-A/-B/-DRB1 haplotypes in donors from Mozambique and corresponding frequencies in the CEDACE Registry. Dataset: **L3F**.

Haplotype	Angola	Global
30 42 03	1.6129%	0.0547%
01 08 03	1.4585%	2.9784%
30 14 15	1.3441%	0.0152%
02 14 01	1.0753%	0.5944%
01 57 07	1.0753%	0.6179%
74 15 13	1.0753%	0.0094%
29 44 07	1.0159%	1.9949%
23 07 15	0.9409%	0.0353%
11 35 01	0.9186%	0.9164%
02 07 15	0.9175%	0.6833%
03 35 15	0.8886%	0.1581%
02 35 11	0.8870%	0.4992%
29 07 15	0.7833%	0.0698%
02 51 03	0.7358%	0.1401%
02 51 13	0.6954%	0.7574%
68 35 13	0.6720%	0.0672%
66 58 13	0.6720%	0.0082%
02 50 07	0.6720%	0.8603%
30 57 13	0.6720%	0.0110%
02 15 07	0.6720%	0.1655%
02 35 15	0.6720%	0.0424%
36 53 11	0.6720%	0.0211%
02 51 15	0.6720%	0.2680%
02 44 04	0.6581%	1.4742%
02 15 03	0.6083%	0.0721%
02 44 13	0.6010%	0.9824%
03 44 07	0.5962%	0.1495%
23 58 13	0.5377%	0.0557%
33 14 01	0.5376%	1.2262%
30 18 03	0.5376%	0.5488%
02 15 11	0.5376%	0.1959%
03 35 11	0.5376%	0.3532%
23 53 13	0.5376%	0.0093%
68 15 09	0.5376%	0.0194%
01 49 04	0.5376%	0.0670%
24 44 13	0.5376%	0.2273%
03 51 13	0.5376%	0.0863%
23 15 01	0.5376%	0.0068%
33 15 03	0.5376%	0.0040%
23 58 15	0.5376%	0.0021%
01 51 04	0.5161%	0.2298%
30 08 15	0.4555%	0.0007%
23 51 13	0.4456%	0.0102%
03 35 01	0.4354%	1.0120%
02 44 07	0.4213%	1.0981%
02 14 13	0.4032%	0.0486%
02 57 13	0.4032%	0.0435%
02 49 04	0.4032%	0.2613%
68 53 11	0.4032%	0.0261%
29 44 01	0.4032%	0.0831%

Supplementary Table 13. 3 *loci*, low-resolution frequencies of the 50 most frequent HLA-A/-B/-DRB1 haplotypes in donors from Angola and corresponding frequencies in the CEDACE Registry.
Dataset: **L3F**.

Supplementary material: 3.2.3.2 Results/HLA frequency analysis/Haplotype frequencies/ In African and foreign donors

Haplotype	Brazil	Global
01 08 03	1.5668%	2.9784%
02 07 15	1.4321%	0.6833%
29 44 07	1.4097%	1.9949%
33 14 01	1.2815%	1.2262%
03 35 01	1.1927%	1.0120%
02 44 07	1.1820%	1.0981%
02 44 13	1.1391%	0.9824%
03 07 15	1.0032%	1.1802%
02 50 07	0.9936%	0.8603%
11 35 01	0.8022%	0.9164%
02 51 07	0.7966%	0.5538%
02 51 13	0.7917%	0.7574%
02 44 04	0.7517%	1.4742%
02 15 11	0.7210%	0.1959%
02 35 13	0.6812%	0.2665%
02 15 04	0.6136%	0.4712%
02 51 11	0.6034%	0.8598%
01 57 07	0.6000%	0.6179%
24 35 11	0.5978%	0.6431%
03 51 11	0.5943%	0.2341%
02 51 08	0.5705%	0.9191%
01 15 13	0.5335%	0.2026%
23 44 07	0.5284%	0.9495%
68 40 04	0.5254%	0.0251%
29 44 15	0.5248%	0.1192%
02 14 01	0.5081%	0.5944%
02 35 08	0.5054%	0.1368%
30 18 03	0.5000%	0.5488%
02 08 03	0.4867%	0.5135%
24 08 03	0.4789%	0.4021%
02 07 01	0.4720%	0.1753%
02 52 15	0.4407%	0.0917%
24 35 13	0.4407%	0.2021%
68 15 03	0.4169%	0.0551%
68 53 13	0.4124%	0.3129%
30 14 13	0.3997%	0.0290%
31 39 08	0.3987%	0.0065%
02 40 13	0.3947%	0.2113%
02 39 04	0.3889%	0.0166%
02 07 11	0.3845%	0.0476%
03 35 11	0.3805%	0.3532%
31 40 04	0.3791%	0.2687%
26 38 13	0.3770%	0.4050%
03 07 11	0.3725%	0.1315%
03 15 13	0.3566%	0.1367%
11 35 07	0.3515%	0.1511%
32 14 07	0.3475%	0.2688%
01 35 01	0.3466%	0.1050%
03 51 01	0.3459%	0.0403%
01 57 04	0.3453%	0.1318%

Supplementary Table 14. 3 *loci*, low-resolution frequencies of the 50 most frequent HLA-A/-B/-DRB1 haplotypes in donors from Brazil and corresponding frequencies in the CEDACE Registry. Dataset: **L3F**.

<u>Supplementary material</u>: 3.2.3.3 Results/HLA frequency analysis/Haplotype frequencies/ Hardy-Weinberg equilibrium

**Dataset**	**Population**	**HLA-A**	**HLA-B**	**HLA-C**	**HLA-DRB1**
L3R	North	**SD**	**SD**		ND
	Center	**SD**	**SD**		**SD**
	MA of Lisbon	**SD**	**SD**		**SD**
	Alentejo	ND	ND		ND
	Algarve	ND	ND		ND
	AR of Madeira	**SD**	ND		ND
	AR of Azores	ND	**SD**		**SD**
L3D	Viana do Castelo	ND	ND		ND
	Braga	ND	**SD**		**SD**
	Vila Real	ND	ND		ND
	Bragança	ND	ND		ND
	Porto	**SD**	**SD**		ND
	Aveiro	ND	**SD**		**SD**
	Viseu	ND	**SD**		ND
	Guarda	ND	**SD**		ND
	Coimbra	ND	**SD**		ND
	Castelo Branco	ND	ND		ND
	Leiria	**SD**	**SD**		**SD**
	Lisboa	**SD**	**SD**		**SD**
	Santarém	**SD**	ND		**SD**
	Portalegre	ND	**SD**		ND
	Setúbal	**SD**	**SD**		**SD**
	Évora	ND	ND		ND
	Beja	ND	ND		ND
	Faro	ND	ND		ND
	Madeira	**SD**	ND		ND
	Açores	ND	**SD**		**SD**
L4R	North	**SD**	ND	ND	**SD**
	Center	**SD**	ND	ND	**SD**
	MA of Lisbon	**SD**	**SD**	**SD**	**SD**
	Alentejo	ND	ND	ND	ND
	Algarve	ND	ND	ND	ND
	AR of Madeira	ND	ND	ND	ND
	AR of Azores	ND	ND	ND	**SD**
L3NW	African	**SD**	**SD**		ND
L3F	Cape Verde	ND	ND		ND
	Mozambique	ND	ND		ND
	Angola	ND	ND		ND
	Brazil	ND	ND		ND

Supplementary Table 15. Deviations from Hardy-Weinberg equilibrium in HLA *loci* of datasets **L3R**, **L3D**, **L4R**, **L3NW** and **L3F**. ND: no deviation; SD: significant deviation.
